# Identification of typical medullary breast carcinoma as a genomic sub-group of basal-like carcinomas, a heterogeneous new molecular entity

**DOI:** 10.1186/bcr1666

**Published:** 2007-04-06

**Authors:** Anne Vincent-Salomon, Nadège Gruel, Carlo Lucchesi, Gaëtan MacGrogan, Remi Dendale, Brigitte Sigal-Zafrani, Michel Longy, Virginie Raynal, Gaëlle Pierron, Isabelle de Mascarel, Corinne Taris, Dominique Stoppa-Lyonnet, Jean-Yves Pierga, Rémy Salmon, Xavier Sastre-Garau, Alain Fourquet, Olivier Delattre, Patricia de Cremoux, Alain Aurias

**Affiliations:** 1 Department of Tumor Biology, Institut Curie, 26 rue d'Ulm, 75248 Paris cedex 05, France; 2 INSERM Unit 830, Institut Curie, 26 rue d'Ulm,75248 Paris cedex 05, France; 3 Translational Research Department, Institut Curie, 26 rue d'Ulm 75248 Paris cedex 05, France; 4 Department of Pathology, Institut Bergonié, 229 Cours de l'Argonne 33076 Bordeaux cedex, France; 5 Department of Radiation Therapy, 26 rue d'Ulm, Institut Curie, 75248 Paris cedex 05, France; 6 Breast Cancer Study Group, Institut Curie, 26 rue d'Ulm, 75248 Paris cedex 05, France; 7 Department of Medical Oncology, Institut Curie, 26 rue d'Ulm 75248 Paris cedex 05, France; 8 Department of Breast Surgery, Institut Curie, 26 rue d'Ulm 75248 Paris cedex 05, France

## Abstract

**Introduction:**

Typical medullary breast carcinoma (MBC) has recently been recognized to be part of the basal-like carcinoma spectrum, a feature in agreement with the high rate of *TP53 *mutations previously reported in MBCs. The present study was therefore designed to identify phenotypic and genetic alterations that distinguish MBCs from basal-like carcinomas (BLC).

**Methods:**

Expression levels of estrogen receptor (ER), progesterone receptor (PR), ERBB2, TP53, cytokeratins (KRTs) 5/6, 14, 8/18, epidermal growth factor receptor and KIT, as well as *TP53 *gene sequence and high-density array comparative genomic hybridization (CGH) profiles, were assessed and compared in a series of 33 MBCs and 26 BLCs.

**Results:**

All tumors were negative for ER, PR and ERBB2. KRTs 5/6 were more frequently expressed in MBCs (94%) than in BLCs (56%) (*p *= 0.0004). *TP53 *mutations were disclosed in 20/26 MBCs (77%) and 20/24 BLCs (83%). Array CGH analysis showed that a higher number of gains (95 regions) and losses (34 regions) was observed in MBCs than in BLCs (36 regions of gain; 13 regions of losses). In addition, gains of 1q and 8q, and losses of X were found to be common to the two groups, whereas gains of 10p (53% of the cases), 9p (30.8% of the cases) and 16q (25.8% of the cases), and losses of 4p (34.8% of the cases), and amplicons of 1q, 8p, 10p and 12p were the genetic alterations found to characterize MBC.

**Conclusion:**

Our study has revealed that MBCs are part of the basal-like group and share common genomic alterations with BLCs, the most frequent being 1q and 8q gains and X losses; however, MBCs are a distinct entity within the basal-like spectrum, characterized by a higher rate of KRT 5/6 expression, a higher rate of gains and losses than BLCs, recurrent 10p, 9p and 16q gains, 4p losses, and 1q, 8p, 10p and 12p amplicons. Our results thus contribute to a better understanding of the heterogeneity in basal-like breast tumors and provide potential diagnostic tools.

## Introduction

Medullary breast carcinomas (MBCs) represent 2% of all invasive breast carcinomas, and although these tumors show aggressive pathological features they are often associated with a more favorable outcome. They are defined by an association of five morphological features [1]: a predominantly syncytial growth pattern, a circumscribed border, a moderate to marked lymphoplasmacytic infiltrate, poorly differentiated nuclear grade with high mitotic rate, and the absence of glandular features or any *in situ *component. Despite these defined morphological features, reproducibility of diagnosis is only moderate. Therefore, more specific and new diagnostic criteria such as genetic features would be very helpful. Gene expression profile analysis classifies breast carcinomas into five groups: luminal–epithelial/estrogen receptor (ER)-positive A and B, basal-like, ERBB2, and normal-like carcinomas [2-5]. The basal-like group of tumors was associated with a poorer outcome than that of luminal tumors [5]. Moreover, basal-like carcinomas (BLCs) were characterized by a specific immunophenotype that was negative for ER, progesterone receptor (PR) and ERBB2, and positive for cytokeratin (KRT) type 5/6, KRT 14 or KRT 17, epidermal growth factor receptor (EGFR) and KIT; [5,6] they were also associated with *TP53 *mutations [3]. We have previously shown that MBCs were associated with a very high rate of *TP53 *mutations [7]. Recently MBCs were found to present a basal-like/myoepithelial phenotype [8,9] with a specific gene overexpression profile [10]. We and others have demonstrated that MBCs have a favorable outcome despite the aggressive pathological features at presentation [11-13] and unlike basal-like tumors [5]. This favorable outcome may be explained in part by the radiosensitivity and chemosensitivity of MBCs [11]. To address the biological differences between MBCs and BLCs, we performed an immunophenotype analysis, a *TP53 *sequence analysis and large-scale analysis by array comparative genomic hybridization (aCGH) on a series of 33 MBCs and compared their immunoprofiles and genetic alterations with those of a group of 26 non-medullary BLCs. These analyses reveal that MBCs have distinct specificities, both at the immunophenotypic level, including more frequent cytokeratin 5/6 expression, and at the genomic level with a high level of chromosome gains and losses, recurrent 10p, 9p and 16q gains and 4p losses, and 1q, 8q, 10p and 12p amplicons.

## Materials and methods

### Patients and tumors

We studied tumors from two groups of patients. Experiments were performed in accordance with Bioethics Law no. 2004-800 and the Ethics Charter from the National Institute of Cancer (INCa). The first group consisted of 33 MBCs; the second group consisted of 26 non-medullary BLCs.

We initially selected 42 patients with MBCs from our files, prospectively registered in our institutions (32 cases from Institut Curie and 10 cases from Institut Bergonié) for which a representative paraffin-block sample and a frozen sample were both available. Samples with less than 50% of tumor cells were excluded from the study. A retrospective pathological review of all cases was performed by four breast pathologists (AVS, BSZ, GMG and IdM) in accordance with Ridolfi's criteria [1], namely pushing margins, syncytial growth pattern without any glandular structures, high nuclear grade with a vesicular chromatin and a high mitotic activity, a dense lymphoplasmacytic infiltrate and no associated ductal carcinoma *in situ*. We have considered here that the three major morphological traits that most clearly distinguish MBCs from BLCs were as follows: first, the presence of large nuclei, irregular in shape, with vesicular chromatin and large eosinophilic nucleoli; second, cells organized in solid syncytial sheets seven to eight cells thick, in a ribbon-like structure; and third, the interspersing of the ribbon-like structure with a stroma composed of a dense lymphoplasmacytic infiltrate with no myofibroblasts and no fibrosis. Seven cases were excluded after pathological review and two for insufficient aCGH quality (see Additional file 1); 33 patients with MBCs were eventually available for genetic analysis.

The second group of tumors were from 26 patients with BLCs. These cases were selected from our files among ER^- ^PR^- ^ERBB2^- ^invasive ductal carcinomas, according to their basal immunophenotype, defined as follows: ER^- ^PR^- ^ERBB2^- ^with the expression of at least one of the following markers: basal-myoepithelial KRT 5/6, KRT14, EGFR and KIT.

### DNA and RNA extraction

DNA was extracted from frozen tumor samples with the use of a standard phenol/chloroform procedure. A frozen tissue section was performed on the remaining frozen fragments to evaluate tumor cellularity. All tumors analyzed presented more than 50% of tumor cells on the control tissue section. Total RNA was extracted from frozen tumor samples with the RNeasy^® ^kit (Qiagen, Courtaboeuf, France), then the RNA clean up kit (Macherey Nagel, France), in accordance with the manufacturers' instructions. The quality of each RNA sample was measured with an Agilent 2100 bioanalyzer. The quantity of RNA was measured by spectrophotometry at 260 nm.

### Immunohistochemistry

Immunostaining was performed on 4 μm tissue sections prepared from a representative sample of the tumor. After rehydration and antigenic retrieval in citrate buffer (10 mM, pH 6.1), tissue sections were stained for ER (clone 6F11, 1/200 dilution; Novocastra, Newcastle Upon Tyne, UK), PR (clone 1A6, 1/200 dilution; Novocastra), ERBB2 (clone CB11, 1/1,000 dilution; Novocastra), EGFR (HER1; clone 31G7, 1/40 dilution; Zymed), CD117 (an epitope of KIT, polyclonal, 1/100 dilution; Dako A/S, Glostrup, Denmark), TP53 (clone DO7, 1/50 dilution; Dako), KRT 5/6 (clone D5/16B4, 1/50 dilution; Dako), KRT 8/18 (clone DC10, 1/100 dilution; Zymed) and KRT 14 (clone LL002, prediluted; Biogenex, San Ramon, CA, USA). Staining was conducted with the Vectastain Elite ABC peroxidase mouse IgG kit (Vector Laboratories, Burlingame, CA, USA), with diaminobenzidine (Dako) as the chromogen. Positive nuclear staining for ER, PR and TP53 was recorded in accordance with standardized guidelines, using 10% of positive cells as the cutoff for ER, PR and TP53. For ERBB2, only staining of membranes was considered, as defined previously [14,15]. CD117 and KRT 5/6, KRT 14 and KRT 8/18 were scored positive if any (weak or strong) cytoplasmic and/or membranous invasive carcinoma cell staining was observed in accordance with previous published studies [6,8,16,17]. For EGFR, 10% of positive membranous staining was used as the positive cutoff. For each antibody, internal and external controls were included in the experiments.

### *TP53 *sequencing

*TP53 *was sequenced in accordance with the previously published procedure [7] or directly from DNA. The *TP53 *sequence was not assessed for nine cases for which no more RNA or DNA was available after aCGH. In brief, for sequencing from RNA, total RNA was reverse-transcribed in a final volume of 20 μl containing 1× reverse transcriptase buffer, 10 units of RNase inhibitor (Promega, Madison, WI, USA), 10 mM dithiothreitol, 50 units of Superscript II RNase H reverse transcriptase (Life Technology, Inc., Cergy Pontoise, France), 1.5 mM random hexamers (Pharmacia, Uppsala, Sweden) and 1 μg of total RNA. Samples were incubated at 20°C for 10 minutes and at 42°C for 30 minutes; reverse transcriptase was then inactivated by heating at 99°C for 5 minutes and cooling for 5 minutes. For sequencing from DNA, total DNA was amplified with primers specific for exons 4 to 10 (primer sequences are listed in Additional file 1). Purified PCR products were sequenced bidirectionally with the use of Big Dye Terminator chemistry (Applied Biosystems, Foster City, CA, USA) with an ABI Prism 3700 DNA Analyzer (Applied Biosystems, Foster City, CA, USA). The central part of the *TP53 *gene (amplified from cDNA between exons 4 and 10, namely codons 42 to 374) was sequenced for all samples.

### Array CGH

The array used contains 3,261 bacterial artificial chromosome (BAC) and P1-derived artificial chromosome (PAC) DNAs, as described previously [18]. All BACs and PACs were verified by end sequencing before spotting, and their positions on the genome have been determined. In brief, after digestion with *Dpn*II (Ozyme, Saint Quentin en Yvelines, France) and column purification (Qiagen PCR Purification Kit; Qiagen), 1.5 μg of tumor DNA and 1.5 μg of normal male DNA were labeled using the BioPrime DNA Labeling System Kit (Invitrogen, Cergy-Pontoise, France), with Cy5dCTP or Cy3-dCTP (Amersham, Uppsala, Sweden), respectively. Labeled tumor and reference DNAs were mixed and precipitated together with 225 μg of human Cot-1 DNA (Invitrogen), resuspended in 65 μl of hybridization buffer (50% formamide, 40 mM NaH_2_PO_4_, 0.1% SDS, 10% dextran sulfate, 2 × standard saline citrate (SSC), Denhardt's solution), denatured, prehybridized for 90 minutes at 37°C and hybridized on treated microarray slides. After 24 hours of hybridization at 37°C in a humidity chamber and appropriate washing, slides were scanned with a GenePix 4000B scanner (Axon Instruments Inc., Union City, CA, USA) and analyzed with GenePix Pro 5.1 image analysis software, which defined the spots and determined the median intensities for the Cy3 and Cy5 signals of each BAC clone.

### Analysis of aCGH data

Data analysis was based on the normalized ratios of Cy5/Cy3 signals observed for each BAC clone [19] that previously passed the flag assessment procedure (see Additional file 2). For autosomal chromosomes, the loss of a given locus was defined by a ratio less than or equal to 0.8, a gain was defined by a ratio greater than or equal to 1.2 and less than 2.0, and an amplicon was defined by a ratio greater than or equal to 2.0. For X chromosomes, a loss was defined by a ratio less than or equal to 1.2, a gain was defined by a ratio greater than or equal to 1.7, and an amplicon was defined by a ratio greater than or equal to 2.5. Whenever a statistical hypothesis concerning the levels of the ratios was tested, the hypothesis was considered to be rejected for *p *values less than or equal to 0.05. Whenever *p *values were adjusted for multiple hypothesis testing, the false discovery rate using the Benjamini–Hochberg procedure was applied (R-Multitest package).

Data analysis was performed with two complementary approaches: a descriptive approach and a statistical approach. For the descriptive approach, the frequency of BAC locus gains, losses and amplicons in the two groups of tumors was evaluated to identify genomic regions that could distinguish MBC tumors from BLC tumors. For a given tumor, a region of gain or loss was defined as a set of at least two consecutive BAC loci meeting the gain and loss criteria described above. A region of gain or loss was considered as recurrent when it was observed in at least 20% of tumors. The median frequency was calculated for each region defined previously, to determine the most recurrent alterations. The regions of gains and losses were compared between the two groups by a Fisher exact test, BAC locus by BAC locus (*p *values were not adjusted for multiple tests). A region was considered to be significantly different between the two groups when the majority of the loci in the region presented *p *values less than 0.05.

For analysis of amplicons, the identification criteria were BAC loci consisting of at least one clone with a ratio greater than or equal to 2.0 in one tumor. Finally, a between-group comparison of the mean ratios of each BAC loci in the two groups of tumors was performed; ratios were log_2 _transformed, the normal distribution of ratios in the two groups was tested by the Shapiro–France test (R-Nortest package), comparisons were performed with a Welch *T *test and the hypothesis of no difference between the two groups was rejected when the Benjamini–Hochberg false discovery rate, calculated over the 3,264 BAC loci analyzed, was less than or equal to 5%.

For the unsupervised statistical approach, hierarchical clustering was performed to define tumor groups with no *a priori *biological knowledge (see Additional file 3). Its robustness was tested by a resampling approach (see Additional file 3). The enrichment of the groups in BLC and MBC tumors was then tested (Welch *T *test), and genomic regions able to discriminate between the two groups were determined.

### Fluorescence *in situ *hybridization (FISH) experiments

To verify the most recurrent gains observed on the aCGH results, FISH analysis was performed on representative frozen samples of four MBCs that demonstrated 10p gains on aCGH, and four BLCs without 10p gains. Frozen tissue sections 4 μm thick were prepared and fixed for 10 minutes in a mixture of ethanol/acetic acid (2:1, by volume). A probe was selected within the regions of gains on the 10p chromosome that differentiated MBC from BLC clusters (TelVysion Spectrum Green 10p from Vysis-chr10: 328,262 to 328,575, and BAC clone g1int953-chr10: 22,497,751 to 22,668,197) and a BAC clone associated with a normal ratio in every case (g1int978-chr10: 50,248,127 to 50,413,107) as control. The BAC DNAs were extracted in accordance with standard procedures. The DNAs were then labeled by nick translation using the BioNick Labeling Kit (BioNick Labeling System; Invitrogen) and the following fluorescent nucleotides: Texas red-dUTP (NEN, Paris, France) for g1int953, and Cy3-dUTP (Amersham, Orsay, France) for g1int978. Each 10p probe was pooled and co-precipitated with the control probe and with an excess of human Cot1 DNA. Denaturation of the probes was performed at 94°C for 10 minutes; the tissue sections were denatured in formamide and 2 × SSC at 72°C for 2 minutes. The probes were then hybridized on the tissue section under a coverslip. Slides were incubated overnight at 37°C in humid chambers, then washed briefly in 1 × SSC for 5 minutes at 20°C and for 5 minutes at 72°C. Finally, slides were counterstained with 4,6-diamidino-2-phenylindole (Vector Laboratories). Images were captured with an Axioplan 2 microscope (Zeiss, Le Pecq, France) with appropriate filters and a Quantix camera (Photometrics, Tucson, AZ, USA) using SmartCapture software (Digital Scientific, Cambridge, UK). Images of an average of 30 nuclei per case were taken and the number of signals for each probe was counted. The mean number of copies of each locus was then determined and compared with the control locus.

## Results

### Clinical characteristics of patients

Because several studies have shown a higher incidence of MBCs in young patients, in particular in a *BRCA1 *context, we compared the ages of the two groups of patients (MBC group: median 53.5 years; range 34 to 76 years; BLC group: median 60 years; range 37 to 82 years; Table 1). The age tended to be younger in MBCs, but the difference was not significant (*p *= 0.072, Wilcoxon rank test). Other clinical characteristics were also noted. The clinical stage was T1 for 14 patients with MBC and 6 with BLC, T2 for 15 patients with MBC and 19 with BLC, T3 for 2 patients with MBC and 1 with BLC, N0 for 24 patients with MBC and 18 with BLC, and N1 for 7 patients with MBC and 8 with BLC (clinical stage was not determined for 2 patients with MBC). All MBCs were of grade III; in comparison, 19 BLCs were of grade III and seven were of grade II (*p *= 0.0056, χ^2 ^test).

### *TP53 *mutations

We have previously reported that *TP53 *mutations were a genetic trait of MBCs [7], and a high rate of such mutations has been found in BLCs [3]. We therefore assessed the *TP53 *sequences in 26 of the 33 MBCs and 24 of the 26 BLCs (Table 1). A mutation in *TP53 *was found in 20 of the 26 MBCs (77%); 16 of these were associated with intense TP53 immunolabeling of tumor cell nuclei (16/20 mutations; Table 2). There was a *TP53 *mutation in 20 of the 24 BLCs sequenced (83%), including five of the seven grade II tumors. In the BLCs, TP53 immunolabeling was concordant with *TP53 *mutation status in all cases (100% of mutated cases). No significant difference in *TP53 *status was observed between MBCs and BLCs (*p *> 0.05).

### Comparative immunophenotypes of MBCs and BLCs

To address the question of whether MBCs and BLCs present any immunophenotypic difference, we analysed the status of markers that contribute to the definition of a basal-like immunophenotype, namely ER, PR, ERBB2, KRT 5/6, KRT 14 and KRT 8/18 in the two groups (Table 2). We observed that all tumors from the MBCs group and the selected BLCs tumors were negative for ER, PR and ERBB2. However, a comparison of the immunophenotype of the two groups of tumors showed that MBCs were more often positive for KRT5/6 than BLCs (30/32 (94%) versus 14/25 (56%), respectively; *p *= 0.0007). No difference in immunophenotype was observed between MBCs and BLCs for KRT14 (17/31 (55%) versus 18/23 (78%); not significant), TP53 (20/33 (61%) versus 12/25 (48%); not significant), KRT 8/18 (30/32 (94%) versus 19/25 (76%); not significant), EGFR (22/32 (69%) versus 10/22 (45%); not significant) or KIT (19/32 (59%) versus 12/20 (60%); not significant).

### Array CGH results

To our knowledge, no unique, standardized method for the analysis of aCGH data has yet been published. We therefore decided to analyse our data with two different approaches: the in the first we looked for the regions already known to be amplified in breast carcinogenesis and described the other new ones. We also evaluated the frequency of gains and losses in the two groups. As a second approach, we used unsupervised clustering. The outcomes of these two approaches led us to draw the same conclusions, which suggest that the new BLC entity could gather different genomic subgroups, one being MBC.

The genes most frequently involved in breast carcinogenesis (*MYC*, *CCND1*, *EGFR *and *CCNE*) were very rarely found amplified in the two groups.

In 17 different MBCs (51%), 59 different amplicons, observed in at least one tumor, were found; in 9 different BLCs (35%), 39 different amplicons were observed. Recurrent amplicons were rare (Table 3; see also Additional file 4).

Amplicons located on chromosomes 1q, 8p, 10p and 12p were within the most frequent regions of gains and were never observed in BLCs. Only amplicons in 8q24, encompassing *MYC*, and in Xq28 were observed in both groups.

**Figure 1:**
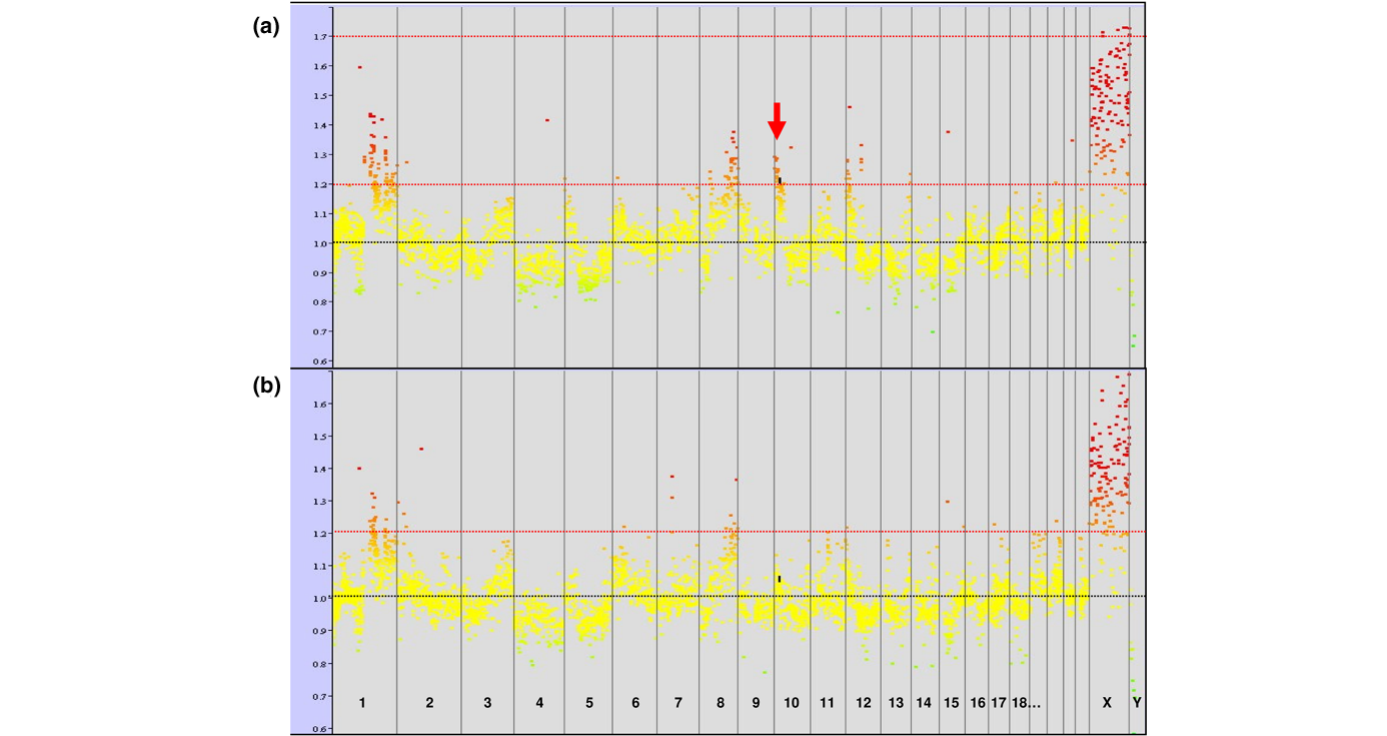
Average genome-wide copy number profiles for medullary breast carcinoma (MBC) and basal-like carcinoma (BLC) tumors. Common alterations observed in both the MBC **(a) **and BLC **(b) **groups are gains of 1q, 8q and Xq (black arrows). The most frequent specific alteration of MBCs (10p gains) is indicated by a red arrow. Ordinates: loci ratios, abscissa: order of the loci on chromosomes from 1 to Y.

The complete list of regions of gains and losses (frequency greater than 20%) in MBCs and BLCs is presented in Additional file 5. The discriminative regions of gains and losses in MBCs and BLCs are listed in Table 4, and an average of the genomic profiles of BLCs and MBCs are presented in Figure 1.

As shown in Table 4, very discriminative regions of gains were observed on chromosomes 3, 9, 10, and 16 in the MBCs. The highest frequencies of gain were observed at 10p13 (53%) (*p *= 0.018). A discriminative region of loss was observed on chromosome 4p15.2 (34.8%) (*p *= 0.025).

BLCs were associated with fewer rearrangements than MBCs (see Additional file 5). One discriminative region of loss was observed on the X chromosome in BLCs, with a median frequency of 38.5% of cases (*p *= 0.001).

Common regions of gains and losses were observed on chromosomes 1q, 8q and Xq in both groups.

We performed a genome-wide comparison of the mean ratios between the MBC and BLC groups. Comparison of the two groups was performed, BAC locus by BAC locus, with a Welch *T *test. This test identified only four differential BAC loci between the two groups, three of them located in chromosome 10 and one in chromosome 12. Only two of the BAC loci of chromosome 10 (coordinates on chr10: 6,669,591 to 6,856,917 and 7,349,041 to 7,496,158, respectively) constituted a contiguous region of gain observed in the MBC group but not in the BLC group.

**Figure 2:**
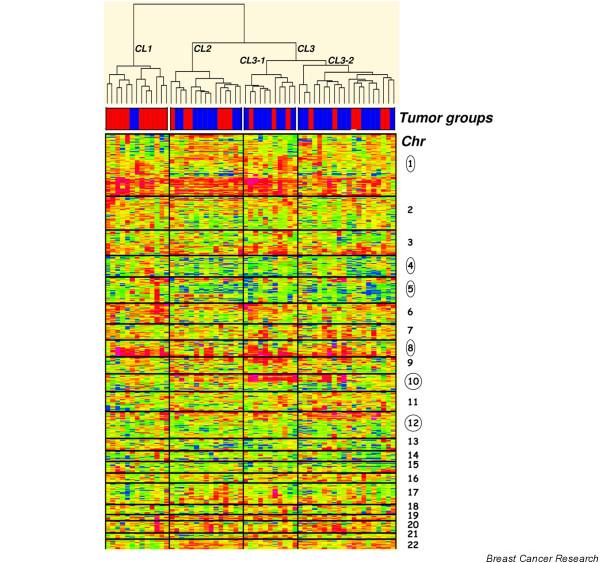
Dendro-heatmaps of the unsupervised clustering by tumors. The standardized copy number log ratio of all autosomal chromosome loci is shown according to a color scale (top). Tumor groups are distinguished with a color code at the bottom of the dendrogram: red, basal-like carcinomas; blue, medullary breast carcinomas.

**Figure 3:**
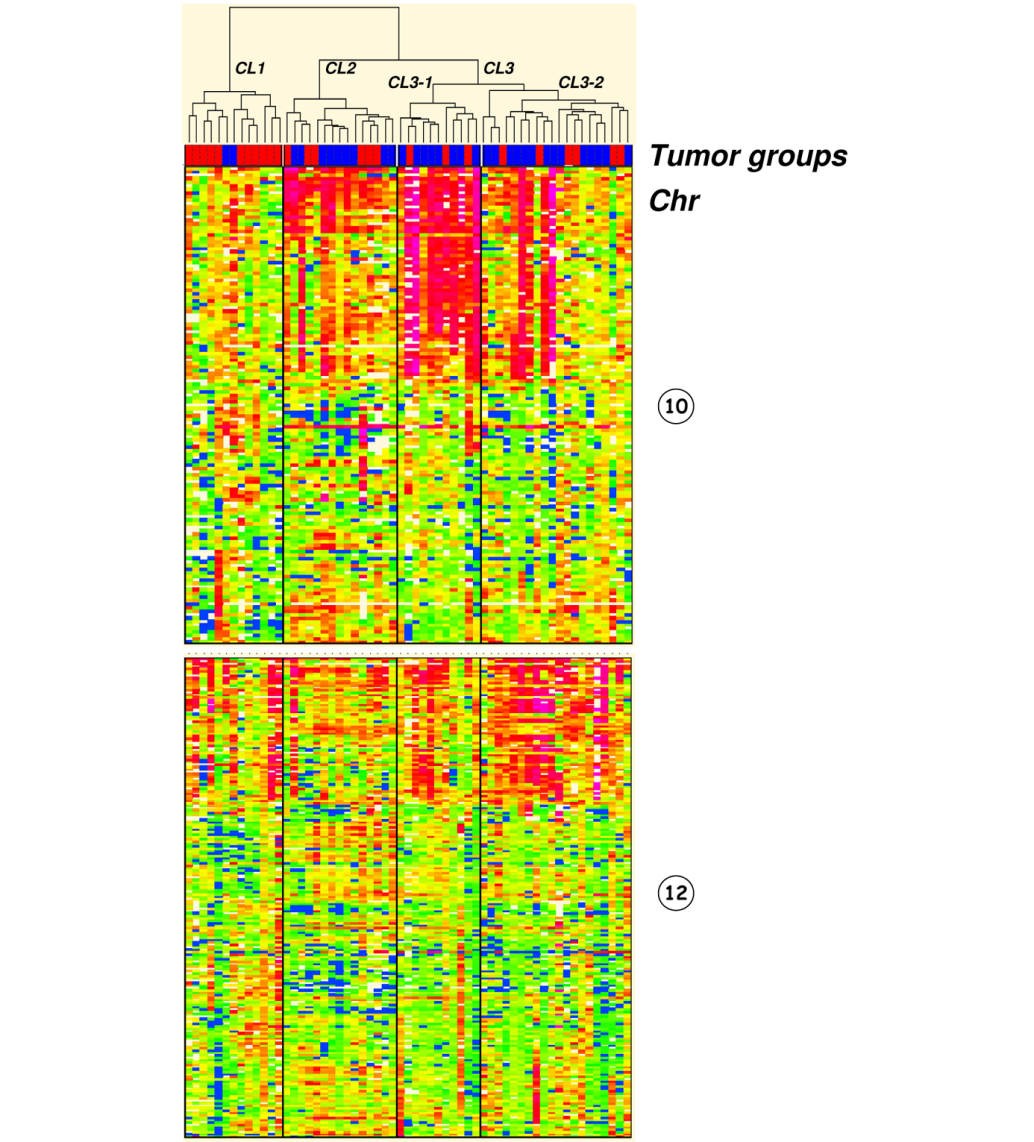
Dendro-heatmaps of chromosomes 10 and 12. Cluster 3-1 shows a more marked gain of the 10p region than in the other clusters. Tumor groups are distinguished with a color code at the bottom of the dendrogram: red, basal-like carcinomas; blue, medullary breast carcinomas.

With unsupervised clustering (Figure 2), three different clusters were identified: cluster 1 composed of 11 BLCs (85%) and 2 MBCs (15%), cluster 2 composed of 6 BLCs (40%) and 9 MBCs (60%), and cluster 3 composed of 9 BLCs (29%) and 22 MBCs (71%). The differences between these clusters were statistically significant (*p *= 0.012). The dendrogram of the tumor ratios in order of genomic coordinates (Figure 2) showed that gain of chromosomes 1q and 8q were common to all tumors. Chromosomes 4 and 5 showed a common trend towards loss. Chromosomes 10 and 12 had a different gain profile depending on the tumor cluster (Figure 3): BLC-enriched cluster 1 had a flat profile (around the normal), and cluster 2 showed a clear gain of distal 10p. The dendrogram identified two subgroups in cluster 3: the first subgroup showed a marked and wide 10p gain profile (cluster 3-1), while the second subgroup (cluster 3-2) might present a 12p gain profile. The robustness of this clustering has been confirmed by three different multiple resampling approaches: the first testing the resampling of tumors (consensus clustering methodology of Monti and colleagues [20]), the second testing the resampling of clones (Multiscale bootstrap methodology of Suzuki and colleagues [21]), and a double leave-one-out methodology developed in the laboratory, testing the resampling of both tumors and clones (Additional file 3).

**Figure 4:**
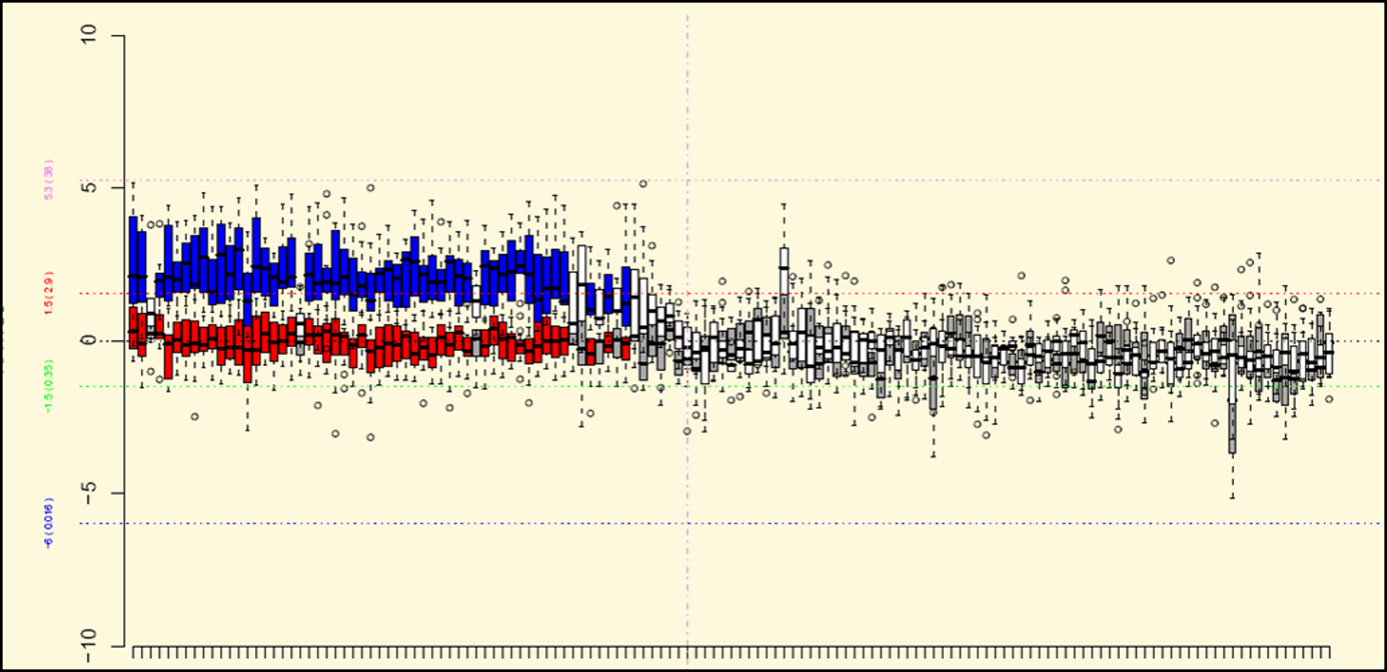
Box plot of the bacterial artificial chromosome (BAC) loci in chromosome 10. The results of the statistical comparison of the mean of ratios between clusters 1 and 3-1 are shown. On this figure, the BAC loci that are significantly different between the two clusters by the Welch *T *test (*p *≤ 0.05, Benjamini–Hochberg corrected for genome-wide multiple test) and that lie outside the calculated fold range in at least one cluster are shown in different colors: red, cluster 1; blue, cluster 3-1. The BAC loci that are not significantly different in terms of *p *value and fold range are shown in grey and white, for cluster 1 and cluster 3-1, respectively. The position of the centromere is shown with a vertical pink dashed line.

A genome-wide comparison of the mean ratios (Welch *T *test) between cluster 1 and cluster 3-1 identified three large discriminative regions of gain encompassing chromosome 10p (Figure 4): the first region was from BAC g11E11 (chr10: 3,723,029 to 3,892,793) to BAC g1int932 (chr10: 14,207,506 to 14,350,672) loci, the second was from BAC g1int936 (chr10: 15,897,438 to 16,092,470) to BAC g1int945 (chr10: 22,556,720 to 22,717,956) loci, and the third was from BAC g1int946 (chr10: 22,559,518 to 22,660,196) to BAC g1int961 (chr10: 24,596,826 to 24,767,011) loci. The second and third regions also differentiated cluster 2 from cluster 3-1 significantly. In contrast, a comparison of clusters 1 and 3-2 did not identify any discriminative regions on chromosome 12 or other chromosomes. Although chromosome 12p seemed to be gained in cluster 3-2 (amplicons excluded), the difference between the two clusters was not statistically significant.

### DNA FISH results on MBC and BLC tumors

We chose to assess whether the 10p gains, observed in 53% of MBCs and discriminant between clusters 1 and 3-1, could be detected by FISH on frozen tissue sections. For this purpose we analyzed four MBCs tumors with a 10p gain and four control BLCs without a 10p gain.

In MBCs, the mean aCGH ratio was 1.8 for the TelVysion SG locus and 1.6 for the g1int 953 locus, for a mean aCGH ratio of 1.0 for the control locus, g1int978. FISH showed mean numbers of 2.7 and 2.4 copies per nucleus for the first two loci, in the presence of 1.6 copies of the g1int978 control locus.

**Figure 5:**
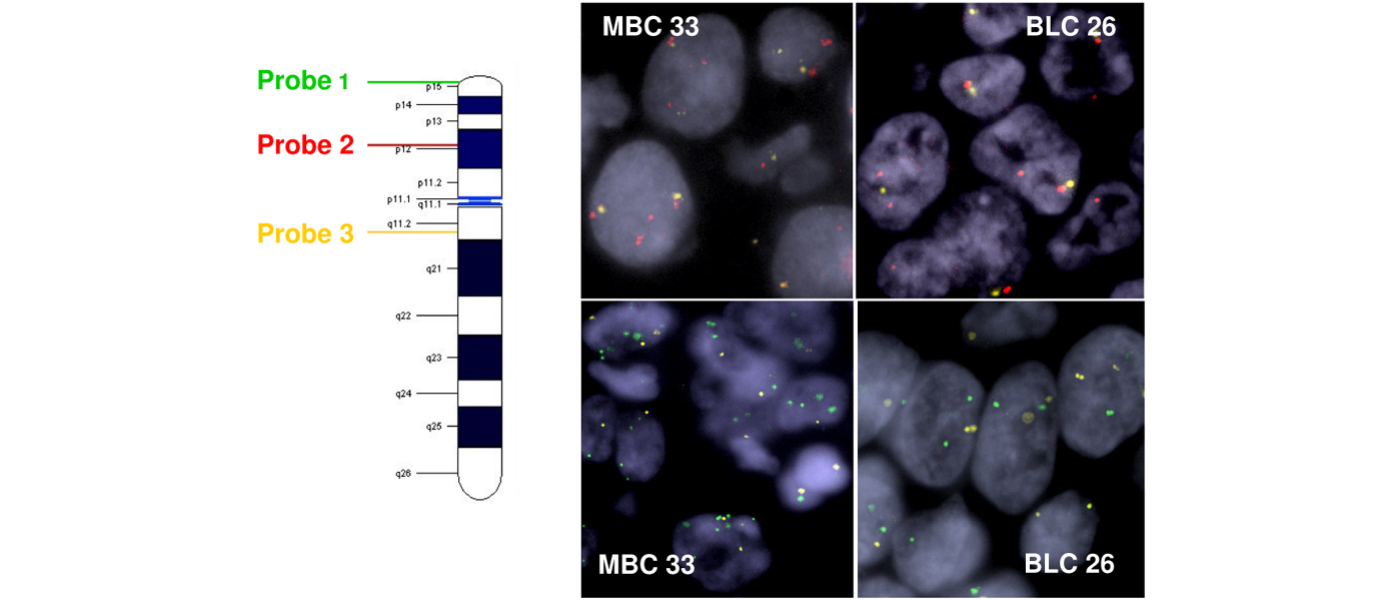
Confirmation of 10p gains with the use of fluorescence *in situ *hybridization (FISH). FISH analysis of cases 33 (medullary breast carcinoma) and 26 (basal-like carcinoma) with TelVysion SG 10p (probe 1) shown in green, g1int953 in red (probe 2), and control probe g1int978 in yellow (probe 3).

In BLCs, the mean aCGH ratio was 0.9 for the TelVysion SG locus, and 1.0 for the g1int 953 locus, for a mean aCGH ratio of 0.9 for the control locus, g1int978. Mean numbers of 1.1 and 1.0 copies per nucleus were observed by FISH for the first two loci, in the presence of 1.4 copies of the g1int978 control locus (Figure 5).

## Discussion

Expression profile analyses [2-4] have categorized invasive breast carcinomas into five groups: luminal A and B, ERBB2^+^/ER^-^, basal-like and normal breast tumors. The basal-like group was subsequently defined, using immunohistochemistry, as being ER^- ^PR^- ^ERBB2^-^, with tumor cells expressing at least one basal-myoepithelial marker such as KRT 5/6 or KRT 14 in association with EGFR or KIT [6,8,22]. Although some BLCs are associated with a favorable outcome [23,24], most tumors with a basal-like immunophenotype have been recognized to be associated with a poor prognosis [5,6,8]. MBCs have recently been shown to share the basal-like immunophenotype [9], and some morphological traits that have been reported to be specific for BLC with poor prognosis, such as tumor necrosis, a pushing border of invasion, and a stromal lymphocytic infiltrate [22,25], are also observed in MBCs. These phenotypic and immunophenotypic similarities suggest that these two entities have a common biology despite the fact that MBC presents a more favorable outcome [11,12]. In the present study we wished to define the possible phenotypic and genetic differences between MBCs and BLCs with a view to understanding the major differences in clinical outcome between these tumors.

Subtle morphological differences between BLCs and MBCs suggest that MBCs could be associated with a specific biology such as the low expression of smooth muscle α-actin in MBCs, corroborating the absence of myofibroblasts in MBC stroma [10]. In our study, we confirmed that MBCs shared some basal-like immunophenotypic features and are ER^- ^PR^- ^ERBB2^- ^tumors but have some specificities such as a more frequent KRT5/6 staining of the cells than in BLCs. Abd El-Rehim and colleagues also previously observed [8] that MBCs expressed KRT 5/6 but not KRT 14. Tot [26] and Jacquemier and colleagues [9] found lower rates of KRT5/6 positivity (25% and 44.7%, respectively) than in the present study, but Tot used a different antibody from that used in the present study and, in contrast with the present study, Jacquemier and colleagues included medullary carcinomas that were ER positive and PR positive in 10.5% and 51.3% of cases, respectively. These differences could explain the higher rates of KRT5/6 staining observed in our series of MBCs than in previously published reports.

We also observed high rates of *TP53 *mutation in the MBC and BLC groups (77% and 83%, respectively). These rates are in agreement with previously reported results for MBCs and BLCs [3,7].

In terms of genetic changes, as described previously by Chin and colleagues [27], in this basal-like spectrum of tumors we found low-level copy-number alterations and infrequent high-level recurrent amplifications. In addition, MBCs seemed to harbor more gains and losses than BLCs. As seven BLCs tumors were of grade II and all MBCs were of grade III, we could not exclude the possibility that this genomic difference could be simply related to grade. However, five of the seven grade II BLCs had a mutation in *TP53*.

As part of the common basal-like spectrum genomic signature, we found that chromosome 8q, including the 8q24 *MYC *region, was frequently gained in MBCs and in BLCs. This result is in agreement with most published data demonstrating that breast carcinomas with *MYC *amplifications were more frequently ER negative [28] or of the medullary type [29]. It has also been reported that *MYC *amplification occurs in younger patients [30]. The same tendency was observed in our MBC series (53.5 years in MBCs compared with 60.0 years in BLCs). Moreover, it has also been shown that *MYC *contributes to tumor progression in *BRCA1*-associated breast cancers and that it was gained or amplified in 53% of cases [31]. In agreement with these data, 3 of the 20 patients with MBC (15%), in whom it was studied, presented a *BRCA1 *mutation (data not shown), which is a higher rate than expected (less than 5%). Taken together, these data suggest that MBC oncogenesis, as part of the basal-like spectrum, could occur more frequently in a context of genetic predisposition than other breast carcinoma types.

Our results suggest that, of the recurrent alterations described for *BRCA1*-associated and high-grade carcinomas, some could be specific for the basal-like spectrum of tumors [23,27,32-34].

In addition, we report the existence of a combination of genetic alterations associated with the MBC phenotype, namely gains of 3q, 9p, 10p and 16q, losses of 4p, and amplicons of 1q, 8p, 10p and 12p. These data document the heterogeneity of the basal-like group of tumors and contribute to the understanding of its carcinogenesis. If confirmed, these alterations would be helpful in improving the relatively poor diagnostic reproducibility for MBCs [35].

Some putative candidate genes linked with oncogenesis, such as *ZMYND11 *(*BS69*), *GATA3*, *CCND2*, *TNFRSF1A *and *BCL2L14*, are present within the two amplicons observed in 10p and 12p. These observations are in agreement with a recent MBC gene expression profile study that showed a gene signature specific for MBCs, with genes from the 12p13 region involved in the same pathways [10].

Previous genetic studies have shown that MBCs, like the basal-like tumor spectrum [27] demonstrated a high rate of chromosome imbalances [36] rather than recurrent high-level amplification. This preferential mechanism of oncogenesis has already been described in predisposition-related cancer syndromes [37]. Our study demonstrates that MBCs effectively presented a high number of gains and losses, suggesting a putative alteration within a DNA damage repair process. If confirmed, this hypothesis could lead to an explanation of their higher sensitivity to radiation therapy and their favorable local and regional outcome [11,12]. These features have already been reported for *BRCA1/2*-related tumors [37,38]. It will therefore be extremely interesting in the future to compare the aCGH profiles of our MBCs with those of *BRCA1*-associated carcinomas. It has also been described previously that an upregulation of cyclin E induces a chromosome instability [39]. Cyclin E overexpression described in MBCs [40] and *BRCA1*-associated tumors [41] could therefore participate in the high chromosomal instability of these tumors.

We also observed that losses in Xq were significantly more frequent in BLCs than in MBCs. These findings are in line with previous publications showing that BLCs display chromosome X abnormalities [42]. Further studies must be conducted to analyze whether the genes involved in the X chromosome alterations correspond to genes from the active or inactive X chromosome.

## Conclusion

We have refined in depth the molecular characteristics of MBCs. MBCs tumors belong to the ER^- ^PR^- ^ERBB2^- ^group, harboring a basal-like immunophenotype with a higher rate of KRT 5/6 positivity than BLCs. Furthermore, MBCs have genetic alterations in common with BLCs, such as frequent *TP53 *mutations, 8q gains or amplification, 1q gains or amplicons, and X amplicons. Nevertheless, we observed that, in addition to a large number of chromosomal alterations, MBCs are characterized by more frequent 10p, 9p, 3q and 16q gains, 4p losses, and 1q, 8p, and 10p and 12p amplicons. Taken together, these data demonstrate that MBCs, although belonging to the spectrum of basal-like tumors, are associated with specific alterations. A better knowledge of the heterogeneity of the basal-like tumor group should be useful in the near future for more appropriate patient management. Furthermore, the new regions that we have defined as being characteristically modified in MBCs should provide insight into the oncogenic features of this particular class of tumors.

## Abbreviations

aCGH = array comparative genomic hybridization; BAC = bacterial artificial chromosome; BLC = basal-like carcinoma; ER = estrogen receptor; FISH = fluorescence *in situ *hybridization; KRT = cytokeratin; MBC = medullary breast carcinoma; PAC = P1-derived artificial chromosome; PR = progesterone receptor; SSC = standard saline citrate.

## Competing interests

The authors declare that they have no competing interests.

## Authors' contributions

AVS and NG participated in the design of the study, retrospective and immunophenotype analysis, performed aCGH, FISH and *TP53 *sequencing, participated in data analysis and drafted the manuscript. CL performed statistical analysis. VR performed aCGH analysis. GP participated in *TP53 *sequencing of BLCs. CT performed immunolabeling. GMG, BSZ and IdM participated in retrospective analysis of the cases. RD, ML, RS, JYP and XSG participated in data collection. AA, OD, PdC, AF, DSL and BSZ participated in the design of the study, data analysis and interpretation of the results. All authors read and approved the final manuscript.

## Supplementary Material

Additional File 1 A file showing forward and reverse primer sequences used for *TP53 *sequencing, exon by exon from exon 4 to exon 10. Click here for file

Additional File 2 A file describing methods used for the raw data analysis of the array CGH experiments, including a description of the quality control criteria. Click here for file

Additional File 3 A file describing the statistical methods used in the data analysis of the array CGH experiments for unsupervised clustering, and the resampling and frequency analyses. Click here for file

Additional File 4 A file showing, for each locus of the array, the normalized CGH ratio. Tumor numbers, identical to those in Table 1, are indicated. Click here for file

Additional File 5 A file showing all the gains and losses, ordered by chromosomes and by coordinates, observed in more than 20% of a given group. The significantly discriminative regions are highlighted in grey. Click here for file

## Figures and Tables

**Table 1:** Clinical and biological characteristics of patients with MBCs and BLCs

Case no.	Age (years)	TNM stage	Histoprognostic grade	*TP53 *status	
BLC					
1	69	T2N0	3	ex7	G→A/p. Arg 248 Gln
2	66	T1N0	2	ex5	G→T/p. Cys 176 Phe
3	60	T2N0	2	ex8	G→A/p. Asp 281 Asn
4	64	T2N0	2	int6	Splice
5	51	T2N1a	3	wt	-
6	62	T1N1a	2	wt	-
7	70	T1N0	3	ex7	G→A/p. Gly 245 Ser
8	37	T1N0	2	ex6	C→T/p. Arg 213 STOP
9	62	T2N0	2	ex6	T→G/p. Ser 215 Arg
10	60	T2N1b	3	ex8	C→T/p. Arg 282 Trp
11	52	T2N1	3	ex6	C→T/p. Arg 213 STOP
12	50	T3N1	3	ex5	G→A/p. Arg 175 His
13	79	T2N0	3	ex5	C→T/p. Ala 159 Val
14	48	T2N0	3	ex10	Del codon 333
15	42	T1N0	3	ex6	Del codon 196
16	52	T2N1b	2	wt	-
17	82	T2N0	3	wt	-
18	46	T2N1a	3	nd	-
19	51	T2N0	3	nd	-
20	81	T2N0	3	ex7	A→G/p. Tyr 236 Cys
21	n.d.	T2N0	3	ex6	T→C/p. Ile 195 Thr
22	40	T1cN0	3	ex6	T→G/p. Leu 194 Arg
23	69	T2N0	3	ex5	G→A/p. Arg 175 His
24	69	T2N0	3	ex5	C→A/p. Tyr 126 STOP
25	55	T2N0	3	ex6	C→T/p. Arg 213 STOP
26	77	T2N1	3	ex7	A→C/p. Thr 253 Pro
MBC					
1	46	T1cN1	3	ex7	Del codon 241
2	34	nd	3	ex7	Del codon 252
3	46	T2N0	3	ex5	G→A/p. Arg 175 His
4	72	T1cN0	3	ex8	G→A/p. Arg 280 Lys
5	39	T3N1	3	wt	-
6	53	T2N0	3	nd	-
7	49	T1cN0	3	nd	-
8	49	T1cN0	3	nd	-
9	33	T2N0	3	nd	-
10	63	T2N0	3	nd	-
11	66	T2N0	3	nd	-
12	50	T1cN1a	3	nd	-
13	57	T1cN0	3	ex7	G→A/p. Arg 248 Gln
14	68	T2N0	3	ex6	C→T/p. Arg 213 STOP
15	61	T2N0	3	ex6	A→G/p. Tyr 220 Cys
16	56	T2N0	3	wt	-
17	58	T2N0	3	ex8	G→A/p. Glu 286 Lys
18	57	T2N0	3	ex8	G→A/p. Cys 275 Tyr
19	62	T2N0	3	ex7	G→A/p. Arg 248 Gln
20	35	T1cN0	3	ex6	A→G/p. His 193 Arg
21	54	T1cN1	3	ex7	G→A/p. Cys 242 Tyr
22	36	T2N1	3	wt	-
23	48	T1cN1	3	ex6	Ins codon 213
24	56	T1cN0	3	ex5	C→A/p. Ala 161 Asp
25	42	T3N0	3	ex6	G→A/p. Val 216 Met
26	43	T2N0	3	ex10	G→T/p.Glu 339 STOP
27	66	T1cN0	3	ex5	Ins codon 125
28	50	T1cN0	3	ex5	G→A/p. Arg 175 His
29	41	T2N0	3	ex5	T→C/p. Ser 127 Pro
30	n.d.	n.d.	3	ex6	A→G/p. Tyr 220 Cys
31	76	T2N0	3	wt	-
32	72	T1cN1	3	wt	-
33	67	T1cN0	3	wt	-

BLC, basal-like carcinoma; MBC, medullary breast carcinoma; n.d., not determined; wt, wild type; del, deletion; ins, insertion; int, intron; ex, exon; p, protein.

**Table 2:** Immunophenotypes of basal-like carcinomas and medullary breast carcinomas

Marker		Medullary breast carcinomas (*n *= 33)	Basal-like carcinomas (*n *= 26)	*p*
ER	+	0 (0)	0 (0)	
	-	33(100)	26 (100)	-
PR	+	0 (0)	0 (0)	
	-	33(100)	26 (100)	-
ERBB2	+	0 (0)	0 (0)	
	-	32 (100)	25 (100)	-
	n.d.	1	1	
TP 53	+	20 (61)	12 (48)	
	-	13 (39)	13 (52)	n.s.
	n.d.	0	1	
KRT 5/6	+	30 (94)	14 (56)	
	-	2 (6)	11 (44)	0.0004
	n.d.	1	1	
KRT 8/18	+	30 (94)	19 (76)	
	-	2 (6)	6 (24)	0.06
	n.d.	1	1	
KRT 14	+	17 (55)	18 (78)	
	-	14 (45)	5 (22)	n.s.
	n.d.	2	3	
EGFR	+	22 (69)	10 (45)	
	-	10 (31)	12 (55)	0.06
	n.d.	1	4	
KIT	+	19 (59)	12 (60)	
	-	13 (41)	8 (40)	n.s.
	n.d.	1	6	

n.d., not determined; n.s., not significant. Numbers in parentheses are percentages.

**Table 3:** Recurrent identical amplicons observed in at least two tumors

Chromosome	Start position^a^	End position^a^	Candidate genes	Maximum ratio	Number of cases	
					
					MBC	BLC
1q21.2–21.3	148.109	150.70	*SNX27*, *FLG*, *S100*, *LRN*	2.2	3	0
8p11.21	40.01	42.35	*MYST3*, *ANK1*	2.9	3	0
8q24	122.64	128.80	*MYC*, *KIAA0196*	2.3	2	2
10p15.3	0.25	1.00	*ZMYND11*	2.3	3	0
12p13.32–13.33	2.46	4.31	*CCND2*	2.8	2	0
12p13.2-13.31	5.16	6.37	*TNFRSF1A*, *NTF3*, *LTBR*, *CD9*	2.7	3	0
12p13.2	7.80	11.7	*PRR4*, *Nanog*, *CD69*, *STELLA/DPPA3*, *PHC1*	3.8	3	0
12p13.2	11.95	12.11	*ETV6*, *BCL2L14*	9.1	3	0
Xp11.22	51.21	53.95	*SSX1*, *SSX2*	2.8	0	2
Xq28	152.66	153.69	*MTCP1*, *TZ*, *TKTL1*, *FLNA*	2.8	3	1

MBC, medullary breast carcinoma; BLC, basal-like carcinoma. ^a^UCSC Genome Browser on Human May 2004 Assembly; positions are given in megabases.

**Table 4:** Regions of gains and losses, significantly different between the MBC and BLC groups

Chromosome	Region	Alteration	Start position (bp)	End position (bp)	MBC	BLC	Median frequency (percentage)	*p*
3	3q27	G	159,935,817	163,800,681	G		30.3	0.049
4	4p15.2-15.1	L	27,441,218	30,744,383	L		34.8	0.025
9	9p24	G	628,053	2,280,507	G		30.3	0.0146
9	9p24	G	3,447,196	7,740,086	G		30.3	0.022
9	9p13	G	36,830,822	40,205,823	G		31.8	0.011
10	10p14-13	G	10,572,971	15,532,695	G		53.0	0.018
16	16q24	G	85,913,563	88,552,226	G		25.8	0.01525
X	Xq12–13	L	66,582,902	71,458,243		L	38.5	0.001

MBC, medullary breast carcinoma; BLC, basal-like carcinoma; G, gain; L, loss. Start and end coordinates of the regions are indicated in bp (May 2004 working draft).

## References

[B1] Ridolfi RL, Rosen PP, Port A, Kinne D, Mike V (1977). Medullary carcinoma of the breast: a clinicopathologic study with 10 year follow-up. Cancer.

[B2] Perou CM, Sorlie T, Eisen MB, van de Rijn M, Jeffrey SS, Rees CA, Pollack JR, Ross DT, Johnsen H, Akslen LA (2000). Molecular portraits of human breast tumours. Nature.

[B3] Sorlie T, Perou CM, Tibshirani R, Aas T, Geisler S, Johnsen H, Hastie T, Eisen MB, van de Rijn M, Jeffrey SS (2001). Gene expression patterns of breast carcinomas distinguish tumor subclasses with clinical implications. Proc Natl Acad Sci USA.

[B4] Sorlie T, Tibshirani R, Parker J, Hastie T, Marron JS, Nobel A, Deng S, Johnsen H, Pesich R, Geisler S (2003). Repeated observation of breast tumor subtypes in independent gene expression data sets. Proc Natl Acad Sci USA.

[B5] van de Rijn M, Perou CM, Tibshirani R, Haas P, Kallioniemi O, Kononen J, Torhorst J, Sauter G, Zuber M, Kochli OR (2002). Expression of cytokeratins 17 and 5 identifies a group of breast carcinomas with poor clinical outcome. Am J Pathol.

[B6] Nielsen TO, Hsu FD, Jensen K, Cheang M, Karaca G, Hu Z, Hernandez-Boussard T, Livasy C, Cowan D, Dressler L (2004). Immunohistochemical and clinical characterization of the basal-like subtype of invasive breast carcinoma. Clin Cancer Res.

[B7] de Cremoux P, Salomon AV, Liva S, Dendale R, Bouchind'homme B, Martin E, Sastre-Garau X, Magdelenat H, Fourquet A, Soussi T (1999). p53 mutation as a genetic trait of typical medullary breast carcinoma. J Natl Cancer Inst.

[B8] Abd El-Rehim DM, Pinder SE, Paish CE, Bell J, Blamey RW, Robertson JF, Nicholson RI, Ellis IO (2004). Expression of luminal and basal cytokeratins in human breast carcinoma. J Pathol.

[B9] Jacquemier J, Padovani L, Rabayrol L, Lakhani SR, Penault-Llorca F, Denoux Y, Fiche M, Figueiro P, Maisongrosse V, Ledoussal V (2005). Typical medullary breast carcinomas have a basal/myoepithelial phenotype. J Pathol.

[B10] Bertucci F, Finetti P, Cervera N, Charafe-Jauffret E, Mamessier E, Adelaide J, Debono S, Houvenaeghel G, Maraninchi D, Viens P (2006). Gene expression profiling shows medullary breast cancer is a subgroup of basal breast cancers. Cancer Res.

[B11] Fourquet A, Vilcoq JR, Zafrani B, Schlienger P, Jullien D, Campana F (1987). Medullary breast carcinoma: the role of radiotherapy as primary treatment. Radiother Oncol.

[B12] Dendale R, Vincent-Salomon A, Mouret-Fourme E, Savignoni A, Medioni J, Campana F, Vilcoq JR, de la Rochefordiere A, Soussi T, Asselain B (2003). Medullary breast carcinoma: prognostic implications of p53 expression. Int J Biol Markers.

[B13] Vu-Nishino H, Tavassoli FA, Ahrens WA, Haffty BG (2005). Clinicopathologic features and long-term outcome of patients with medullary breast carcinoma managed with breast-conserving therapy (BCT). Int J Radiat Oncol Biol Phys.

[B14] Vincent-Salomon A, Mac Grogan G, Couturier J, Arnould L, Mathoulin-Pélissier S (2003). Re: HER2 testing in the real world. J Natl Cancer Inst.

[B15] Vincent-Salomon A, MacGrogan G, Couturier J, Arnould L, Denoux Y, Fiche M, Jacquemier J, Mathieu MC, Penault-Llorca F, Rigaud C (2003). Calibration of immunohistochemistry for assessment of HER2 in breast cancer: Results of the French multicentric GEFPICS study. Histopathology.

[B16] Dorfman DM, Bui MM, Tubbs RR, Hsi ED, Fitzgibbons PL, Linden MD, Rickert RR, Roche PC (2006). The CD117 immunohistochemistry tissue microarray survey for quality assurance and interlaboratory comparison: a College of American Pathologists Cell Markers Committee Study. Arch Pathol Lab Med.

[B17] Fulford LG, Easton DF, Reis-Filho JS, Sofronis A, Gillett CE, Lakhani SR, Hanby A (2006). Specific morphological features predictive for the basal phenotype in grade 3 invasive ductal carcinoma of breast. Histopathology.

[B18] Idbaih A, Marie Y, Pierron G, Brennetot C, Hoang-Xuan K, Kujas M, Mokhtari K, Sanson M, Lejeune J, Aurias A (2005). Two types of chromosome 1p losses with opposite significance in gliomas. Ann Neurol.

[B19] Hupe P, Stransky N, Thiery JP, Radvanyi F, Barillot E (2004). Analysis of array CGH data: from signal ratio to gain and loss of DNA regions. Bioinformatics.

[B20] Monti S, Tamayo P, Mesirov J, Golub T (2003). Consensus clustering: a resampling method for class discovery and visualization of gene expression microarray data. Machine Learning.

[B21] Suzuki R, Shimodaira H (2006). Pvclust: an R package for assessing the uncertainty in hierarchical clustering. Bioinformatics.

[B22] Livasy CA, Karaca G, Nanda R, Tretiakova MS, Olopade OI, Moore DT, Perou CM (2006). Phenotypic evaluation of the basal-like subtype of invasive breast carcinoma. Mod Pathol.

[B23] Jones C, Ford E, Gillett C, Ryder K, Merrett S, Reis-Filho JS, Fulford LG, Hanby A, Lakhani SR (2004). Molecular cytogenetic identification of subgroups of grade III invasive ductal breast carcinomas with different clinical outcomes. Clin Cancer Res.

[B24] Azoulay S, Lae M, Freneaux P, Merle S, Al Ghuzlan A, Chnecker C, Rosty C, Klijanienko J, Sigal-Zafrani B, Salmon R (2005). KIT is highly expressed in adenoid cystic carcinoma of the breast, a basal-like carcinoma associated with a favorable outcome. Mod Pathol.

[B25] Tsuda H, Tani Y, Weisenberger J, Kitada S, Hasegawa T, Murata T, Tamai S, Hirohashi S, Matsubara O, Natori T (2005). Frequent KIT and epidermal growth factor receptor overexpressions in undifferentiated-type breast carcinomas with 'stem-cell-like' features. Cancer Sci.

[B26] Tot T (2000). The cytokeratin profile of medullary carcinoma of the breast. Histopathology.

[B27] Chin K, DeVries S, Fridlyand J, Spellman PT, Roydasgupta R, Kuo WL, Lapuk A, Neve RM, Qian Z, Ryder T (2006). Genomic and transcriptional aberrations linked to breast cancer pathophysiologies. Cancer Cell.

[B28] Loo LW, Grove DI, Williams EM, Neal CL, Cousens LA, Schubert EL, Holcomb IN, Massa HF, Glogovac J, Li CI (2004). Array comparative genomic hybridization analysis of genomic alterations in breast cancer subtypes. Cancer Res.

[B29] Al-Kuraya K, Schraml P, Torhorst J, Tapia C, Zaharieva B, Novotny H, Spichtin H, Maurer R, Mirlacher M, Kochli O (2004). Prognostic relevance of gene amplifications and coamplifications in breast cancer. Cancer Res.

[B30] Aulmann S, Adler N, Rom J, Helmchen B, Schirmacher P, Sinn HP (2006). C-myc amplifications in primary breast carcinomas and their local recurrences. J Clin Pathol.

[B31] Grushko TA, Dignam JJ, Das S, Blackwood AM, Perou CM, Ridderstrale KK, Anderson KN, Wei M-J, Adams AJ, Hagos FG (2004). MYC is amplified in BRCA1-associated breast cancers. Clin Cancer Res.

[B32] Melchor L, Alvarez S, Honrado E, Palacios J, Barroso A, Diez O, Osorio A, Benitez J (2005). The accumulation of specific amplifications characterizes two different genomic pathways of evolution of familial breast tumors. Clin Cancer Res.

[B33] Jonsson G, Naylor TL, Vallon-Christersson J, Staaf J, Huang J, Ward MR, Greshock JD, Luts L, Olsson H, Rahman N (2005). Distinct genomic profiles in hereditary breast tumors identified by array-based comparative genomic hybridization. Cancer Res.

[B34] Wessels LF, van Welsem T, Hart AA, van't Veer LJ, Reinders MJ, Nederlof PM (2002). Molecular classification of breast carcinomas by comparative genomic hybridization: a specific somatic genetic profile for BRCA1 tumors. Cancer Res.

[B35] Longacre TA, Ennis M, Quenneville LA, Bane AL, Bleiweiss IJ, Carter BA, Catelano E, Hendrickson MR, Hibshoosh H, Layfield LJ (2006). Interobserver agreement and reproducibility in classification of invasive breast carcinoma: an NCI breast cancer family registry study. Mod Pathol.

[B36] Osin P, Lu YJ, Stone J, Crook T, Houlston RS, Gasco M, Gusterson BA, Shipley J (2003). Distinct genetic and epigenetic changes in medullary breast cancer. Int J Surg Pathol.

[B37] van Beers EH, van Welsem T, Wessels LFA, Li Y, Oldenburg RA, Devilee P, Cornelisse CJ, Verhoef S, Hogervorst FBL, van't Veer LJ, Nederlof PM (2005). Comparative genomic hybridization profiles in human BRCA1 and BRCA2 breast tumors highlight differential sets of genomic aberrations. Cancer Res.

[B38] Kirova YM, Stoppa-Lyonnet D, Savignoni A, Sigal-Zafrani B, Fabre N, Fourquet A (2005). Risk of breast cancer recurrence and contralateral breast cancer in relation to BRCA1 and BRCA2 mutation status following breast-conserving surgery and radiotherapy. Eur J Cancer.

[B39] Spruck CH, Won KA, Reed SI (1999). Deregulated cyclin E induces chromosome instability. Nature.

[B40] Berglund P, Stighall M, Jirstrom K, Borgquist S, Sjolander A, Hedenfalk I, Landberg G (2005). Cyclin E overexpression obstructs infiltrative behavior in breast cancer: a novel role reflected in the growth pattern of medullary breast cancers. Cancer Res.

[B41] Foulkes WD, Brunet JS, Stefansson IM, Straume O, Chappuis PO, Begin LR, Hamel N, Goffin JR, Wong N, Trudel M (2004). The prognostic implication of the basal-like (Cyclin E^high^/p27^low^/p53^+^/glomeruloid-microvascular-proliferation^+^) phenotype of BRCA1-related breast cancer. Cancer Res.

[B42] Richardson AL, Wang ZC, De Nicolo A, Lu X, Brown M, Miron A, Liao X, Iglehart JD, Livingston DM, Ganesan S (2006). X chromosomal abnormalities in basal-like human breast cancer. Cancer Cell.

