# Supplementary material for: Identification of typical medullary breast carcinoma as a genomic sub-group of basal-like carcinomas, a heterogeneous new molecular entity
Source: Breast Cancer Res. 2007 Apr 6;9(2):R24. doi: 10.1186/bcr1666 (PMC1868916; doi:10.1186/bcr1666)
Supplement: Additional File 2 — A file describing methods used for the raw data analysis of the array CGH experiments, including a description of the quality control criteria. [file bcr1666-S2.doc]

**Annex 2: Statistical analysis of CGH array data**

The VAMP and MANOR algorithms[1, 2] were used to perform within-array normalization and correction of spatial effects; BAC loci that did not meet quality criteria were flagged and the normalized Cy5/Cy3 signal ratio of each unflagged BAC loci was calculated for each array. Finally, the among-array and within-array distribution of the flagged BAC loci were analyzed starting from the original set of 3,922 BAC loci and 61 tumor arrays. 59 arrays with less than 20% of their BAC loci flagged were included in the analysis after elimination of 2 tumors from the initial set. Similarly, 3264 clones that were flagged in less than 20% of tumors were conserved in the analysis.

1. La Rosa P, Viara E, Hupe P, Pierron G, Liva S, Neuvial P, Brito I, Lair S, Servant N, Robine N, Manie E, Brennetot C, Janoueix-Lerosey I, Raynal V, Gruel N, Rouveirol C, Stransky N, Stern MH, Delattre O, Aurias A, Radvanyi F, Barillot E. **VAMP: visualization and analysis of array-CGH, transcriptome and other molecular profiles.***Bioinformatics*. 2006, 22:2066-73

2. Neuvial P, Hupe P, Brito I, Liva S, Manie E, Brennetot C, Radvanyi F, Aurias A, Barillot E: **Spatial normalization of array-CGH data**. *BMC Bioinformatics* 2006, **7**:264.
