# Supplementary material for: Identification of typical medullary breast carcinoma as a genomic sub-group of basal-like carcinomas, a heterogeneous new molecular entity
Source: Breast Cancer Res. 2007 Apr 6;9(2):R24. doi: 10.1186/bcr1666 (PMC1868916; doi:10.1186/bcr1666)
Supplement: Additional File 3 — A file describing the statistical methods used in the data analysis of the array CGH experiments for unsupervised clustering, and the resampling and frequency analyses. [file bcr1666-S3.pdf]

**Annex 3: unsupervised clustering and statistical comparison of groups**

All data were initially log2 transformed. The ratio of each BAC locus of the sex chromosomes was standardized so that the median of the BAC loci distribution among tumors was centered on 1. We then verified that the between-tumor correlation for the BAC loci of the X and Y-chromosomes was not significantly different from that generated *in silico* for Gaussian noise (data not shown). We concluded that the BAC loci of the sex chromosomes could be removed from the analysis without impairing the conclusions of the study. The BAC loci ratios of the non-sex chromosomes were then standardized within-tumor by subtracting the median ratio of the tumor and dividing by the median absolute deviation of the same tumor. The gain and loss BAC loci ratio thresholds were recalculated as the interval of values of the ratios in all tumors that covered 95% of their distribution (-1.5 for losses and +1.5 for gains). The threshold for amplicons was the 99$^{th}$ centile of the distribution of ratios (+5.25). Amplicon ratios were then removed before clustering.

For unsupervised clustering, the standard correlation between tumors was chosen as the criterion to define genomic-wide similarity among tumors; the Ward linkage method was used as the criterion to aggregate tumors into groups that maximize their pair-wise correlation[1]. Unsupervised clustering identified 4 groups of tumors (CL1, CL2, CL3-1, CL3-2). In order to assess the robustness of the 4 groups when the composition of genomic loci and tumor samples was modified, we removed one chromosome and one tumor at a time and recalculated the clustering dendrograms (1298 combinations using the same clustering parameters). At each time, we assessed the agreement between the 4 original groups CL1, CL2, CL3-1, CL3-2 and the high level groups S1, S2, S3 and S4 of the new dendrogram. Agreement was tested via the KAPPA techniques (package IRR in R), by counting the number of tumors hold in each original group which were found in the recalculated clustering groups (independent on the ordering of the groups, see Table 1 Annex 3). When the KAPPA

index was greater than or equal to 0.9, the two cluster dendrograms had very high degree of resemblance and we assumed that the removal of the specific chromosome and tumor did not affect the original clustering configuration. The removal of chromosome 3, 16, 19, 20, 21 and 22 did not affect clustering in combination with 50% to 60% of tumors (see Table 1 of Annex 3). The remaining chromosomes were instead critical to maintaining the original clustering configuration. Next, we removed chromosome 3, 16, 19, 20, 21 and 22 and performed a new clustering on the whole set of tumors. As expected, this latest dendrogram showed 4 high levels groups, which were mostly overlapping the original CL1 (black boxes), CL2 (blue boxes), CL3-1 (magenta boxes) and CL3-2 (orange boxes) clusters. In specific the corresponding CL1' and CL3-1' were significantly enriched in BLC and MBC (respectively 88% and 66%, p:0.01). Figure 1 of Annex 3 shows the overlap between the groups of the two clustering.

We then used two already published techniques, the MultiScale Bootstrap Resampling (Suzuki and Shimodaira, 2006) and the Consensus Clustering Resampling (Monti et al., 2003) as an alternative approach to estimate the stability of the clusters.

In order to test the Multiscale Boostrap Resampling methodology, we used the *"pvclust"* and *"scaleboot"* packages in R version 4.1.0 (Suzuki and Shimodaira, 2006). Following the authors' indications, we chose the number of clones of the experiment as the scaling dimension of the bootstrap algorithm; clones were scaled from half to one an a half the size of the original data set (i.e. 3264 clones). Tumors were not resampled. The Approximately Unbiased probability-values for each dendrogram of the original cluster were estimated using the "*scaleboot*" package (Shimodaira H, 2006a R package technical notes). The algorithm calculates the Akaike information criterion (AIC) of goodness of fit for a specific set of numeric approximation methods (Shimodaira H, 2006b). Then following the indications of the author, we used the third order accuracy probability-values which minimized the AIC

mesure. We ran the algorithm with an increasing number of bootstrap resamples (respectively 1,000, 5,000, 10,000, 100,000 runs) to check the convergence of the procedure.

The Approximately Unbiased probability values of our four major clusters are shown in red on the right hand side of each dendrogram in fig 2 of Annex 3. Cluster CL1, CL3-1 and CL3-2, surrounded by a red box, had probability-values higher than the standard 95% confidence threshold (respectively 99%, 99%, 100%). Cluster CL2, surrounded by a blue box, was estimated at a lower 82% probability-value. This is consistent with our original conclusion regarding the robustness of the Basal-enriched and Medullar-enriched clusters (respectively CL1 and CL3-1, CL3-2) whereas CL2 represents a more heterogeneous group of tumors.

To test the Consensus Clustering Resampling methodology (Monti et al. 2003), we coded a script in R following the guidelines in their manuscript. Following the author suggestion, the varying dimension was the number of tumors involved in each resampling cycle. This method is based on the Consensus Matrix that records the number of times any given couple of samples are assigned to the same cluster divided by the total number of times both items are selected by the resampling schema. Resampling without replacement was performed with sub-samples size values varying from 99% to 80% of the tumors (higher limit 58 tumors, lower limit 47 tumors). In all cases 10,000 resampling runs were executed. The set of Consensus Matrix and Cluster Consensus indicators for the different rates of resampling are shown in figure 3 of Annex 3, where colors from white to red represent the values of the consensus index going from 0 to 1.

The data show that the Cluster Consensus Index values of clusters CL1, CL2, CL3-1 and CL3-2 with 95% of the tumors (56) were respectively 0.70, 0.48, 0.83 and 0.22, with 90% of the tumors (53) were 0.62, 0.43, 0.74 and 0.20 and with 85% of the tumors (50) were 0.60, 0.41, 0.72 and 0.19. As expected the Cluster Consensus index took into account the bias due

to the different size of the clusters. Hence CL1 and CL3-1 stood out as the relatively more robust clusters even at lower rate of resampling.

Following, the standard Welch T-test was then used to determine the genomic regions that differentiated the clusters of tumors. Briefly, the normality of each BAC loci log-ratio was tested in each of the 4 groups using the Shapiro-Francia normality test (R-Normtest package). The null hypothesis was then tested for each group and each BAC:

$$H_0 : \mu_{b,c} = 0 \qquad \text{b=1::3,264, c=\{cl1, cl2, cl3-1, cl3-2\}}$$

versus the alternative hypothesis:

$$H_1 : \mu_{b,c} > 0 \quad \text{or} \quad H_1 : \mu_{b,c} < 0$$

where b is a BAC locus, c is one of the 4 clusters and $\mu_{b,c}$ represents the mean ratio of the BAC locus b in cluster c. Hence the mean ratio of each BAC locus was considered significantly different from the theoretical parity of 1 (i.e. the absence of genomic anomaly at the BAC locus) when the False Discovery Rate of the test was less than or equal to 0.05 after Benjamini-Hochberg correction for multiple tests, over the total number of BAC loci[2]. The subset of contiguous BAC loci for which the mean ratio was significantly different from 1 in p-value and falling outside the fold threshold range for deletions and for gains was defined as "candidate" genomic regions of gain/loss in each tumor group. For the BAC loci contained in the "candidate" BAC loci regions, we further tested whether any subsets that could differentiate each cluster from the others by testing the null hypothesis:

$$H_0 : \mu_{b,c} = \mu_{b,g} \qquad \text{b=1::3,264, c, g in \{cl1, cl2, cl3-1, cl3-2\}}$$

versus the alternative hypothesis:

$$H_1 : \mu_{b,c} > \mu_{b,g} \quad \text{or} \quad H_1 : \mu_{b,c} < \mu_{b,g}$$

where b is a BAC locus, c and g are two among the 4 clusters and $\mu_{b,c}$ and $\mu_{b,g}$ represents the mean ratio of the BAC locus b in clusters c and g. The two mean ratios were considered

significantly different when the False Discovery Rate of the test was less than or equal to 0.05 after Benjamini-Hochberg correction for multiple tests over the total number of BACs. The subset of contiguous BAC loci whose mean ratios were significantly different in p-value and falling outside the fold threshold range in at least one of the two groups was considered to constitute "differential" genomic regions of gain/loss of each tumor group.

References


1. **Ward J: Hierarchical grouping to optimize an objective function. *J Am Statis Assoc* 1963, 58:236-244.**
2. **Benjamini Y, Hochberg Y: Controlling the false discovery rate: a practical and powerfull approach to multiple testing. *Journal of the Royal Statistical Society* 1995, 57:289-300.**

3. **Suzuki R, Shimodaira H.: Pvclust: an R package for assessing the uncertainty in hierarchical clustering. Bioinformatics (2006) 22(12):1540-1542**

4. **Shimodaira H: Multiscale bootstrap using scaleboot package. R SCALEBOOT package notes, (2006a), 6-8**

5. **Shimodaira H: Approximately Unbiased Tests for Singular Surfaces via Multiscale Bootstrap Resampling. Research Report B-430, Dept. Mathematical and Computing Sciences, Tokyo Institute of Technology, Tokyo, (2006b)**

6. **Monti S, Tamayo P, Mesirov J and Golub T: Consensus Clustering: A Resampling Method for Class Discovery and Visualization of Gene Expression Microarray Data. Machine Learning (2003), Vol 52 pp91-118**

MEDULLAR vs BASAL (59) LEAVE ONE OUT TEST by CHROMOSOME and TUMOR

K AGREEMENT INDEX BETWEEN CLUSTERING(4grps) OBTAINED without ONE CHROMOSOME/ONE TUMOR and the CLUSTERING OBTAINED with ALL CHROMOSOMEs and ALL TUMORs

| | BLC2 | BLC4 | BLC8 | BLC24 | MBC29 | MBC19 | MBC32 | MBC33 | MBC18 | MBC20 | MBC3 | BLC14 | BLC1 | BLC15 | BLC7 | BLC23 | BLC25 | MBC19 | MBC21 | MBC24 | MBC26 | MBC16 | MBC22 | MBC10 | MBC31 | BLC3 | BLC9 | MBC27 | MBC7 | BLC5 | BLC6 | BLC12 | MBC11 | MBC12 | MBC14 | BLC13 | MBC4 | MBC1 | MBC23 | BLC17 | BLC18 | MBC25 | BLC26 | BLC10 | BLC11 | BLC16 | MBC9 | BLC19 | MBC13 | BLC20 | BLC21 | MBC17 | MBC5 | MBC8 | MBC2 | BLC22 | MBC6 | MBC28 | MBC15 | |
|---|---|---|---|---|---|---|---|---|---|---|---|---|---|---|---|---|---|---|---|---|---|---|---|---|---|---|---|---|---|---|---|---|---|---|---|---|---|---|---|---|---|---|---|---|---|---|---|---|---|---|---|---|---|---|---|---|---|---|---|---|
| 1 | 0.70 | 0.41 | 0.70 | 0.66 | 0.67 | 0.65 | 0.77 | 0.39 | 0.57 | 0.65 | 0.40 | 0.68 | 0.54 | 0.40 | 0.40 | 0.77 | 0.67 | 0.54 | 0.67 | 0.69 | 0.68 | 0.67 | 0.66 | 0.37 | 0.69 | 0.36 | 0.65 | 0.26 | 0.37 | 0.66 | 0.66 | 0.40 | 0.32 | 0.37 | 0.64 | 0.48 | 0.40 | 0.26 | 0.36 | 0.40 | 0.37 | 0.77 | 0.67 | 0.37 | 0.68 | 0.76 | 0.64 | 0.64 | 0.64 | 0.76 | 0.37 | 0.37 | 0.64 | 0.76 | 0.40 | 0.37 | 0.40 | 0.26 | 0.77 | 0 |
| 4 | 0.80 | 0.77 | 0.74 | 0.46 | 0.50 | 0.68 | 0.70 | 0.80 | 0.69 | 0.48 | 0.73 | 0.76 | 0.68 | 0.74 | 0.74 | 0.72 | 0.74 | 0.77 | 0.82 | 0.74 | 0.80 | 0.71 | 0.76 | 0.45 | 0.82 | 0.77 | 0.84 | 0.76 | 0.45 | 0.84 | 0.83 | 0.73 | 0.45 | 0.45 | 0.68 | 0.83 | 0.48 | 0.77 | 0.80 | 0.48 | 0.80 | 0.70 | 0.80 | 0.80 | 0.39 | 0.80 | 0.80 | 0.45 | 0.80 | 0.80 | 0.80 | 0.68 | 0.77 | 0.63 | 0.83 | 0.80 | 0.79 | 0.80 | 0.72 | 0 |
| 5 | 0.72 | 0.74 | 0.81 | 0.77 | 0.74 | 0.79 | 0.70 | 0.76 | 0.52 | 0.79 | 0.77 | 0.76 | 0.72 | 0.81 | 0.70 | 0.81 | 0.74 | 0.73 | 0.66 | 0.79 | 0.79 | 0.76 | 0.65 | 0.26 | 0.80 | 0.78 | 0.86 | 0.85 | 0.79 | 0.79 | 0.79 | 0.77 | 0.74 | 0.26 | 0.79 | 0.75 | 0.28 | 0.79 | 0.79 | 0.28 | 0.71 | 0.79 | 0.79 | 0.84 | 0.84 | 0.79 | 0.79 | 0.74 | 0.79 | 0.70 | 0.79 | 0.79 | 0.81 | 0.80 | 0.79 | 0.26 | 0.79 | 0.81 | 0.84 | 0 |
| 8 | 0.74 | 0.78 | 0.76 | 0.77 | 0.40 | 0.68 | 0.67 | 0.70 | 0.77 | 0.43 | 0.75 | 0.55 | 0.44 | 0.52 | 0.74 | 0.40 | 0.77 | 0.76 | 0.77 | 0.51 | 0.80 | 0.47 | 0.79 | 0.50 | 0.77 | 0.75 | 0.75 | 0.37 | 0.76 | 0.75 | 0.75 | 0.78 | 0.50 | 0.50 | 0.50 | 0.78 | 0.76 | 0.76 | 0.80 | 0.52 | 0.50 | 0.40 | 0.76 | 0.75 | 0.46 | 0.76 | 0.50 | 0.50 | 0.50 | 0.76 | 0.76 | 0.76 | 0.76 | 0.76 | 0.72 | 0.50 | 0.76 | 0.69 | 0.74 | 0 |
| 10 | 0.75 | 0.71 | 0.73 | 0.84 | 0.78 | 0.79 | 0.78 | 0.76 | 0.64 | 0.66 | 0.71 | 0.60 | 0.60 | 0.67 | 0.65 | 0.63 | 0.64 | 0.68 | 0.79 | 0.64 | 0.79 | 0.63 | 0.79 | 0.47 | 0.64 | 0.68 | 0.70 | 0.79 | 0.79 | 0.69 | 0.69 | 0.60 | 0.74 | 0.58 | 0.79 | 0.60 | 0.75 | 0.69 | 0.79 | 0.62 | 0.79 | 0.76 | 0.63 | 0.79 | 0.83 | 0.79 | 0.79 | 0.79 | 0.79 | 0.79 | 0.79 | 0.79 | 0.75 | 0.79 | 0.69 | 0.79 | 0.68 | 0.79 | 0.79 | 0 |
| 12 | 0.56 | 0.52 | 0.65 | 0.38 | 0.40 | 0.56 | 0.55 | 0.52 | 0.58 | 0.37 | 0.37 | 0.74 | 0.31 | 0.41 | 0.65 | 0.39 | 0.31 | 0.57 | 0.38 | 0.36 | 0.61 | 0.39 | 0.55 | 0.38 | 0.38 | 0.65 | 0.66 | 0.32 | 0.38 | 0.37 | 0.37 | 0.41 | 0.38 | 0.38 | 0.39 | 0.75 | 0.76 | 0.39 | 0.39 | 0.40 | 0.38 | 0.40 | 0.57 | 0.31 | 0.80 | 0.38 | 0.38 | 0.38 | 0.38 | 0.39 | 0.38 | 0.38 | 0.65 | 0.38 | 0.33 | 0.38 | 0.79 | 0.40 | 0.37 | 0 |
| 13 | 0.78 | 0.43 | 0.68 | 0.65 | 0.74 | 0.34 | 0.46 | 0.76 | 0.76 | 0.44 | 0.43 | 0.53 | 0.55 | 0.48 | 0.79 | 0.36 | 0.75 | 0.41 | 0.79 | 0.73 | 0.77 | 0.64 | 0.73 | 0.46 | 0.74 | 0.72 | 0.79 | 0.73 | 0.36 | 0.65 | 0.65 | 0.89 | 0.36 | 0.36 | 0.38 | 0.65 | 0.73 | 0.74 | 0.73 | 0.73 | 0.46 | 0.74 | 0.76 | 0.66 | 0.74 | 0.46 | 0.73 | 0.73 | 0.36 | 0.73 | 0.54 | 0.74 | 0.73 | 0.29 | 0.72 | 0.73 | 0.50 | 0.64 | 0.41 | 0 |
| 15 | 0.71 | 0.71 | 0.85 | 0.89 | 0.71 | 0.76 | 0.72 | 0.85 | 0.72 | 0.71 | 0.68 | 0.58 | 0.63 | 0.67 | 0.63 | 0.64 | 0.72 | 0.82 | 0.72 | 0.79 | 0.82 | 0.83 | 0.72 | 0.83 | 0.72 | 0.83 | 0.69 | 0.72 | 0.83 | 0.83 | 0.71 | 0.85 | 0.80 | 0.83 | 0.83 | 0.83 | 0.83 | 0.83 | 0.82 | 0.83 | 0.83 | 0.80 | 0.81 | 0.83 | 0.87 | 0.83 | 0.83 | 0.83 | 0.83 | 0.83 | 0.83 | 0.83 | 0.83 | 0.83 | 0.71 | 0.83 | 0.81 | 0.83 | 0.73 | 0 |
| 2 | 0.83 | 0.71 | 0.79 | 0.78 | 0.69 | 0.76 | 0.78 | 0.69 | 0.78 | 0.54 | 0.71 | 0.76 | 0.72 | 0.79 | 0.86 | 0.69 | 0.68 | 0.68 | 0.78 | 0.27 | 0.76 | 0.68 | 0.78 | 0.43 | 0.68 | 0.67 | 0.79 | 0.71 | 0.53 | 0.83 | 0.83 | 0.86 | 0.25 | 0.69 | 0.27 | 0.93 | 0.68 | 0.73 | 0.80 | 0.30 | 0.69 | 0.69 | 0.60 | 0.78 | 0.79 | 0.53 | 0.68 | 0.68 | 0.68 | 0.68 | 0.68 | 0.68 | 0.68 | 0.68 | 0.67 | 0.69 | 0.78 | 0.78 | 0.80 | 1 |
| 6 | 0.79 | 0.77 | 0.82 | 0.76 | 0.66 | 0.80 | 0.74 | 0.62 | 0.74 | 0.75 | 0.84 | 0.79 | 0.69 | 0.75 | 0.72 | 0.73 | 0.74 | 0.73 | 0.81 | 0.81 | 0.79 | 0.74 | 0.83 | 0.75 | 0.77 | 0.73 | 0.91 | 0.79 | 0.75 | 0.76 | 0.75 | 0.75 | 0.70 | 0.75 | 0.52 | 0.72 | 0.75 | 0.75 | 0.75 | 0.77 | 0.75 | 0.73 | 0.75 | 0.81 | 0.49 | 0.71 | 0.75 | 0.75 | 0.75 | 0.75 | 0.75 | 0.75 | 0.73 | 0.75 | 0.75 | 0.75 | 0.75 | 0.75 | 0.75 | 1 |
| 7 | 0.86 | 0.79 | 0.66 | 0.82 | 0.60 | 0.88 | 0.64 | 0.75 | 0.76 | 0.78 | 0.71 | 0.85 | 0.88 | 0.80 | 0.76 | 0.78 | 0.81 | 0.73 | 0.76 | 0.75 | 0.88 | 0.67 | 0.76 | 0.88 | 0.76 | 0.76 | 0.85 | 0.88 | 0.88 | 0.88 | 0.88 | 0.68 | 0.32 | 0.88 | 0.32 | 0.85 | 0.88 | 0.76 | 0.88 | 0.88 | 0.88 | 0.77 | 0.88 | 0.88 | 0.80 | 0.88 | 0.88 | 0.88 | 0.88 | 0.88 | 0.63 | 0.88 | 0.88 | 0.88 | 0.76 | 0.88 | 0.85 | 0.69 | 0.92 | 1 |
| 9 | 0.83 | 0.72 | 0.88 | 0.77 | 0.69 | 0.79 | 0.67 | 0.80 | 0.65 | 0.69 | 0.84 | 0.65 | 0.60 | 0.79 | 0.73 | 0.69 | 0.65 | 0.79 | 0.77 | 0.41 | 0.77 | 0.77 | 0.71 | 0.77 | 0.71 | 0.77 | 0.90 | 0.72 | 0.77 | 0.77 | 0.77 | 0.80 | 0.72 | 0.77 | 0.77 | 0.80 | 0.77 | 0.81 | 0.77 | 0.77 | 0.77 | 0.37 | 0.64 | 0.83 | 0.79 | 0.77 | 0.77 | 0.77 | 0.77 | 0.77 | 0.77 | 0.77 | 0.77 | 0.77 | 0.77 | 0.77 | 0.77 | 0.77 | 0.72 | 1 |
| 14 | 0.77 | 0.77 | 0.82 | 0.77 | 0.74 | 0.85 | 0.73 | 0.77 | 0.81 | 0.74 | 0.78 | 0.57 | 0.96 | 0.89 | 0.75 | 0.74 | 0.88 | 0.82 | 0.88 | 0.83 | 0.85 | 0.77 | 0.88 | 0.77 | 0.85 | 0.74 | 0.81 | 0.83 | 0.54 | 0.78 | 0.77 | 0.75 | 0.49 | 0.77 | 0.54 | 0.75 | 0.77 | 0.80 | 0.79 | 0.77 | 0.77 | 0.77 | 0.77 | 0.67 | 0.89 | 0.83 | 0.77 | 0.77 | 0.83 | 0.77 | 0.77 | 0.77 | 0.80 | 0.77 | 0.77 | 0.77 | 0.80 | 0.77 | 0.77 | 1 |
| 11 | 0.83 | 0.74 | 0.79 | 0.63 | 0.44 | 0.71 | 0.73 | 0.70 | 0.72 | 0.68 | 0.65 | 0.62 | 0.70 | 0.74 | 0.58 | 0.34 | 0.72 | 0.67 | 0.72 | 0.77 | 0.74 | 0.62 | 0.67 | 0.70 | 0.69 | 0.75 | 0.75 | 0.70 | 0.51 | 0.70 | 0.70 | 0.74 | 0.54 | 0.70 | 0.83 | 0.70 | 0.76 | 0.70 | 0.70 | 0.63 | 0.70 | 0.70 | 0.62 | 0.70 | 0.83 | 0.68 | 0.70 | 0.70 | 0.70 | 0.70 | 0.70 | 0.70 | 0.60 | 0.54 | 0.70 | 0.70 | 0.92 | 0.92 | 0.82 | 2 |
| 17 | 0.89 | 0.77 | 0.74 | 0.76 | 0.66 | 0.77 | 0.72 | 0.80 | 0.82 | 0.79 | 0.84 | 0.88 | 0.50 | 0.83 | 0.79 | 0.76 | 0.83 | 1.00 | 0.83 | 0.89 | 0.79 | 0.74 | 0.83 | 0.70 | 0.77 | 0.83 | 0.83 | 0.76 | 0.83 | 0.81 | 0.83 | 0.96 | 0.83 | 0.83 | 0.83 | 0.89 | 0.83 | 0.83 | 0.79 | 0.83 | 0.83 | 0.80 | 0.71 | 0.81 | 0.83 | 0.83 | 0.83 | 0.83 | 0.83 | 0.83 | 0.83 | 0.83 | 0.83 | 0.83 | 0.83 | 0.83 | 0.75 | 0.83 | 0.83 | 2 |
| 18 | 0.77 | 0.74 | 0.74 | 0.77 | 0.35 | 0.71 | 0.72 | 0.80 | 0.75 | 0.71 | 0.75 | 0.53 | 0.78 | 0.82 | 0.69 | 0.77 | 0.75 | 0.74 | 0.82 | 0.77 | 0.79 | 0.74 | 0.82 | 0.39 | 0.75 | 0.74 | 0.75 | 0.92 | 0.74 | 0.74 | 0.74 | 0.75 | 0.74 | 0.74 | 0.74 | 0.88 | 0.74 | 0.74 | 0.79 | 0.74 | 0.74 | 0.96 | 0.74 | 0.67 | 1.00 | 0.74 | 0.74 | 0.66 | 0.74 | 0.74 | 0.74 | 0.74 | 0.74 | 0.74 | 0.74 | 0.74 | 0.74 | 0.74 | 0.74 | 3 |
| 20 | 0.89 | 0.77 | 0.84 | 0.89 | 0.58 | 0.77 | 0.72 | 0.84 | 0.77 | 0.78 | 0.84 | 0.71 | 0.78 | 0.82 | 1.00 | 0.82 | 0.59 | 0.76 | 0.83 | 0.73 | 0.97 | 0.74 | 0.83 | 1.00 | 0.83 | 1.00 | 0.88 | 0.74 | 0.69 | 1.00 | 1.00 | 0.96 | 0.95 | 0.81 | 1.00 | 0.92 | 1.00 | 0.82 | 0.74 | 1.00 | 1.00 | 0.55 | 0.71 | 1.00 | 1.00 | 1.00 | 1.00 | 1.00 | 1.00 | 1.00 | 1.00 | 1.00 | 1.00 | 1.00 | 1.00 | 1.00 | 0.62 | 1.00 | 1.00 | 28 |
| 19 | 0.89 | 0.74 | 0.73 | 0.76 | 0.80 | 0.80 | 0.72 | 0.73 | 0.77 | 0.82 | 0.77 | 0.76 | 0.75 | 0.82 | 0.79 | 0.79 | 0.77 | 0.73 | 0.83 | 0.82 | 0.79 | 0.74 | 0.83 | 0.77 | 1.00 | 1.00 | 0.74 | 0.93 | 0.76 | 1.00 | 1.00 | 0.96 | 0.74 | 1.00 | 0.99 | 0.74 | 1.00 | 1.00 | 1.00 | 1.00 | 1.00 | 0.97 | 1.00 | 1.00 | 1.00 | 0.99 | 1.00 | 1.00 | 1.00 | 1.00 | 1.00 | 1.00 | 1.00 | 1.00 | 1.00 | 1.00 | 0.99 | 1.00 | 1.00 | 31 |
| 22 | 0.87 | 0.74 | 0.82 | 0.76 | 0.58 | 0.79 | 0.73 | 0.74 | 0.83 | 0.82 | 0.83 | 0.65 | 0.78 | 0.82 | 0.79 | 0.82 | 0.77 | 0.73 | 0.83 | 0.73 | 0.71 | 0.74 | 0.99 | 0.70 | 0.83 | 0.99 | 0.86 | 0.89 | 0.99 | 0.99 | 0.99 | 0.96 | 0.99 | 0.99 | 0.99 | 0.77 | 0.99 | 0.99 | 0.99 | 0.99 | 0.99 | 0.79 | 0.99 | 0.99 | 0.99 | 0.99 | 0.99 | 0.99 | 0.99 | 0.99 | 0.99 | 0.99 | 0.99 | 0.99 | 0.99 | 0.99 | 0.99 | 1.00 | 0.99 | 31 |
| 3 | 0.85 | 0.83 | 0.72 | 0.85 | 0.64 | 0.80 | 0.67 | 0.72 | 0.83 | 0.79 | 0.88 | 0.92 | 0.89 | 0.93 | 0.89 | 0.82 | 0.91 | 0.67 | 0.86 | 0.75 | 0.83 | 0.93 | 0.79 | 0.93 | 0.92 | 0.85 | 0.90 | 0.89 | 0.93 | 0.85 | 0.85 | 0.93 | 0.96 | 0.93 | 0.93 | 0.90 | 0.86 | 0.96 | 0.96 | 0.86 | 0.88 | 0.93 | 0.92 | 0.93 | 0.86 | 0.93 | 0.93 | 0.93 | 0.93 | 0.93 | 0.93 | 0.93 | 0.92 | 0.93 | 0.93 | 0.96 | 0.92 | 0.93 | 0.93 | 32 |
| 16 | 0.89 | 0.86 | 0.84 | 0.89 | 0.36 | 0.80 | 0.71 | 0.84 | 0.83 | 0.61 | 0.77 | 0.80 | 0.78 | 0.82 | 0.79 | 0.90 | 0.83 | 0.83 | 0.83 | 1.00 | 0.79 | 0.67 | 0.83 | 0.69 | 0.83 | 0.79 | 1.00 | 0.97 | 1.00 | 1.00 | 1.00 | 0.67 | 0.95 | 1.00 | 0.69 | 0.90 | 1.00 | 1.00 | 1.00 | 1.00 | 1.00 | 0.97 | 0.99 | 1.00 | 1.00 | 1.00 | 1.00 | 1.00 | 1.00 | 1.00 | 1.00 | 1.00 | 1.00 | 1.00 | 1.00 | 1.00 | 0.99 | 1.00 | 1.00 | 33 |
| 21 | 0.89 | 0.86 | 0.84 | 0.89 | 0.62 | 0.80 | 0.72 | 0.73 | 0.83 | 0.82 | 0.77 | 0.76 | 0.50 | 0.82 | 0.79 | 0.79 | 0.77 | 0.83 | 1.00 | 0.73 | 0.80 | 0.75 | 0.99 | 1.00 | 1.00 | 1.00 | 0.88 | 0.93 | 1.00 | 1.00 | 1.00 | 0.87 | 1.00 | 1.00 | 1.00 | 0.92 | 1.00 | 1.00 | 1.00 | 1.00 | 1.00 | 0.97 | 0.99 | 1.00 | 1.00 | 1.00 | 1.00 | 1.00 | 1.00 | 1.00 | 1.00 | 1.00 | 1.00 | 1.00 | 1.00 | 1.00 | 0.99 | 1.00 | 1.00 | 36 |
| TOT | 0 | 0 | 0 | 0 | 0 | 0 | 0 | 0 | 0 | 0 | 0 | 1 | 1 | 1 | 1 | 1 | 1 | 1 | 1 | 1 | 1 | 1 | 2 | 3 | 3 | 4 | 4 | 4 | 4 | 5 | 5 | 5 | 5 | 5 | 5 | 5 | 5 | 5 | 5 | 5 | 5 | 5 | 5 | 6 | 6 | 6 | 6 | 6 | 6 | 6 | 6 | 6 | 6 | 6 | 6 | 6 | 6 | 7 | 7 | |

RED ====> KAPPA INDEX >= 0.9 =====> REMOVAL of the couple [CHR, TUMOR] DOES NOT AFFECT THE CLUSTERING SHAPE

YEL ====> KAPPA INDEX < 0.9 =====> REMOVAL of the couple [CHR, TUMOR] SIGNIFICANTLY CHANGE THE CLUSTERING SHAPE

# Figure 1 of annex 3

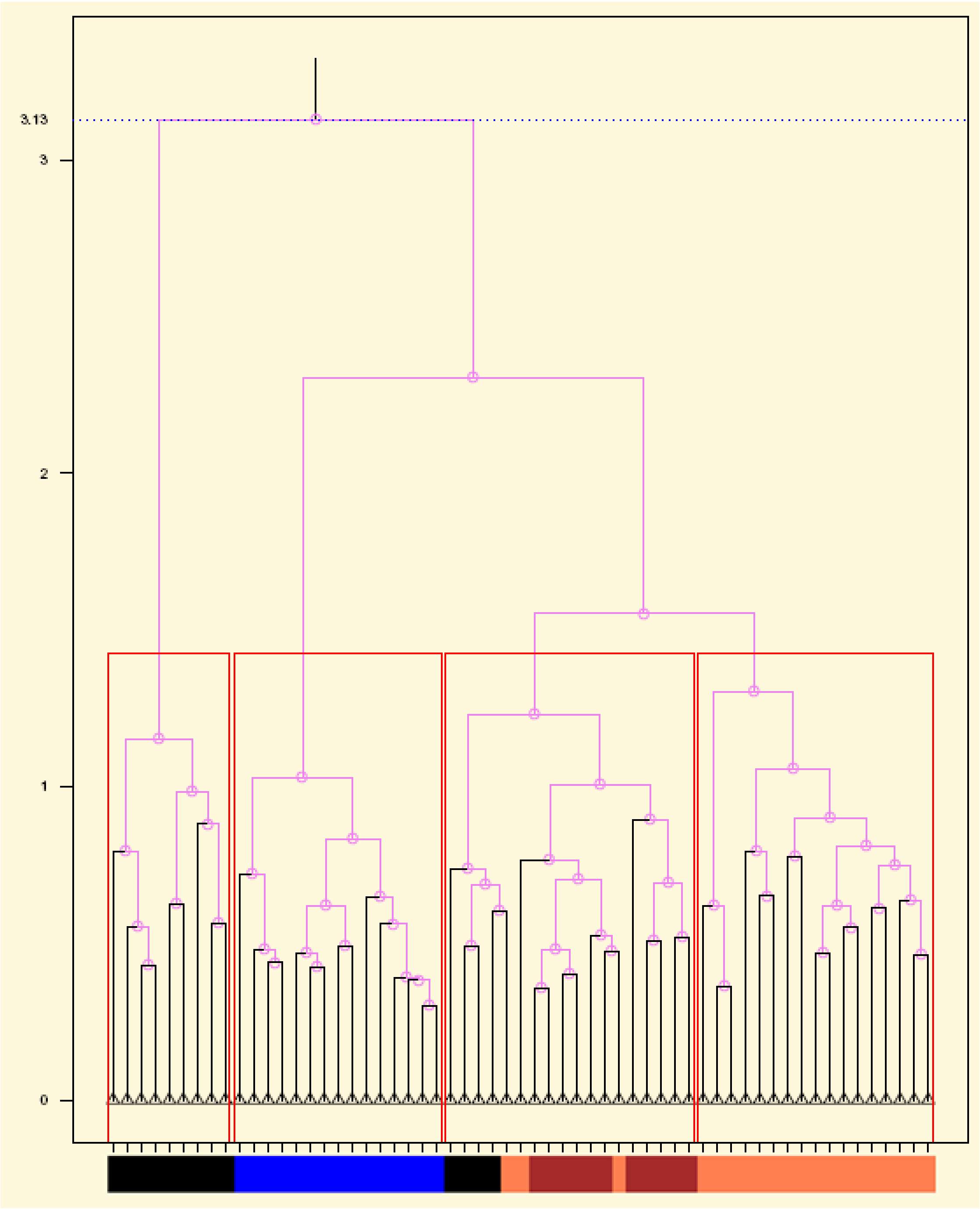

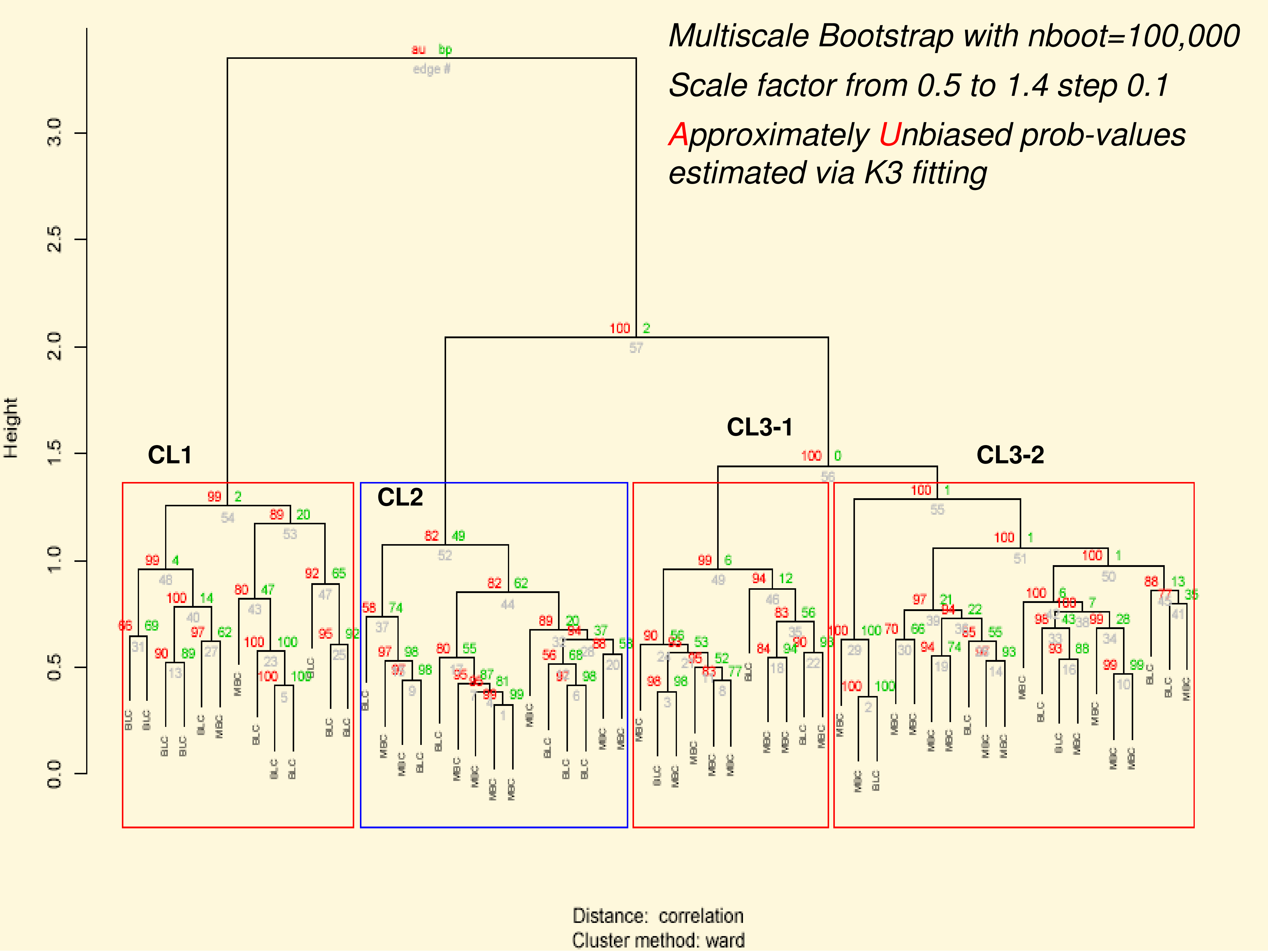

Multiscale Bootstrap with nboot=100,000
Scale factor from 0.5 to 1.4 step 0.1
Approximately Unbiased prob-values
estimated via K3 fitting
au
bp
edge #
Height
CL1
CL2
CL3-1
CL3-2
Distance: correlation
Cluster method: ward

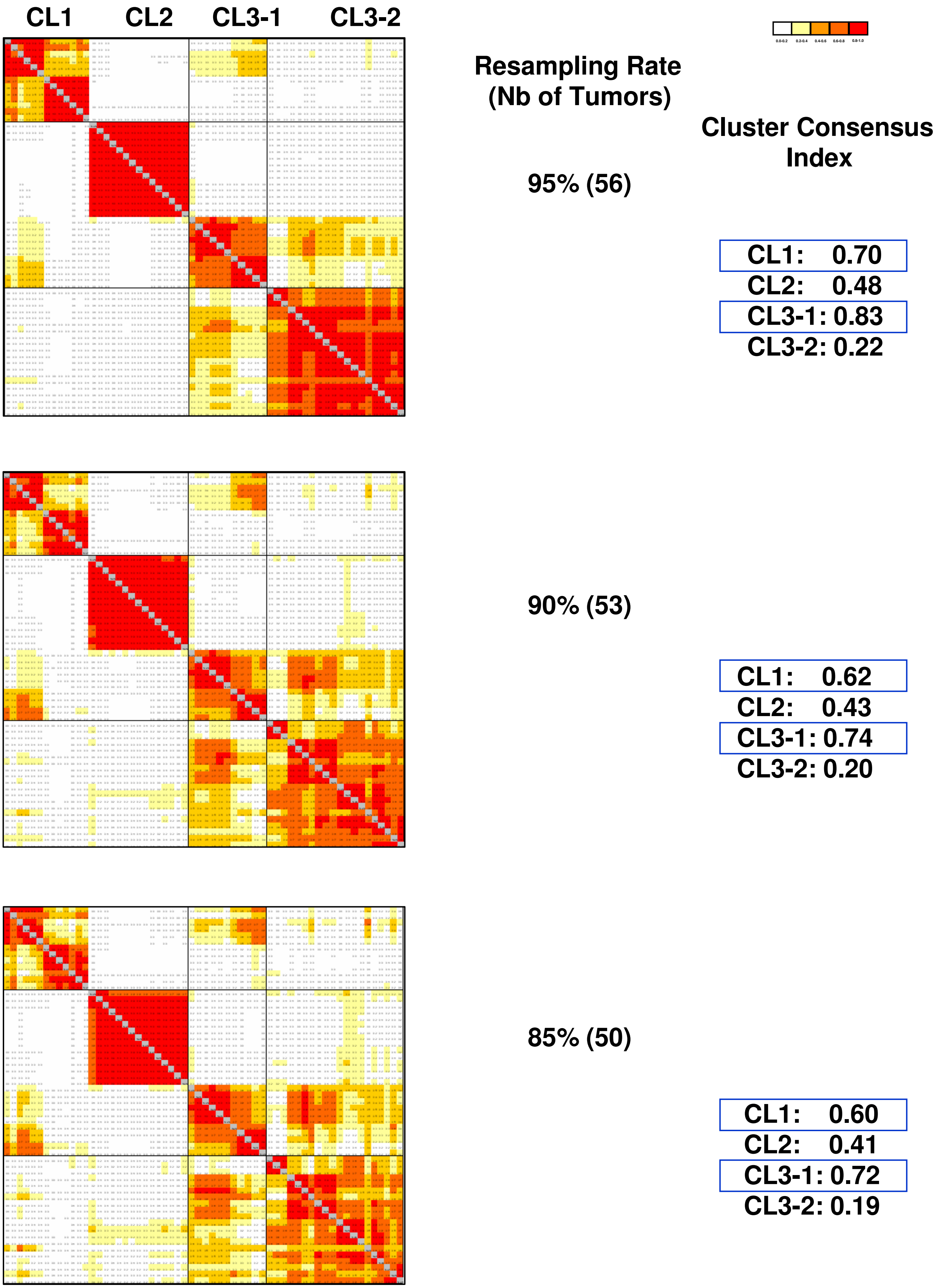
CL1
CL2
CL3-1
CL3-2
Resampling Rate
(Nb of Tumors)
Cluster Consensus
Index
95% (56)
CL1: 0.70
CL2: 0.48
CL3-1: 0.83
CL3-2: 0.22
90% (53)
CL1: 0.62
CL2: 0.43
CL3-1: 0.74
CL3-2: 0.20
85% (50)
CL1: 0.60
CL2: 0.41
CL3-1: 0.72
CL3-2: 0.19
